# Multilevel and Quasi Monte Carlo Methods for the Calculation of the Expected Value of Partial Perfect Information

**DOI:** 10.1177/0272989X211026305

**Published:** 2021-07-07

**Authors:** Wei Fang, Zhenru Wang, Michael B. Giles, Chris H. Jackson, Nicky J. Welton, Christophe Andrieu, Howard Thom

**Affiliations:** Mathematical Institute, University of Oxford, Oxford, Oxfordshire, UK; Mathematical Institute, University of Oxford, Oxford, Oxfordshire, UK; Mathematical Institute, University of Oxford, Oxford, Oxfordshire, UK; MRC Biostatistics Unit, University of Cambridge, Cambridge, Cambridgeshire, UK; Population Health Science, Bristol Medical School, University of Bristol, Bristol, UK; School of Mathematics, University of Bristol, Bristol, UK; Population Health Science, Bristol Medical School, University of Bristol, Bristol, UK

**Keywords:** expected value of partial perfect information, nested expectations, multilevel Monte Carlo, quasi Monte Carlo

## Abstract

The expected value of partial perfect information (EVPPI) provides an upper bound on the value of collecting further evidence on a set of inputs to a cost-effectiveness decision model. Standard Monte Carlo estimation of EVPPI is computationally expensive as it requires nested simulation. Alternatives based on regression approximations to the model have been developed but are not practicable when the number of uncertain parameters of interest is large and when parameter estimates are highly correlated. The error associated with the regression approximation is difficult to determine, while MC allows the bias and precision to be controlled. In this article, we explore the potential of quasi Monte Carlo (QMC) and multilevel Monte Carlo (MLMC) estimation to reduce the computational cost of estimating EVPPI by reducing the variance compared with MC while preserving accuracy. We also develop methods to apply QMC and MLMC to EVPPI, addressing particular challenges that arise where Markov chain Monte Carlo (MCMC) has been used to estimate input parameter distributions. We illustrate the methods using 2 examples: a simplified decision tree model for treatments for depression and a complex Markov model for treatments to prevent stroke in atrial fibrillation, both of which use MCMC inputs. We compare the performance of QMC and MLMC with MC and the approximation techniques of generalized additive model (GAM) regression, Gaussian process (GP) regression, and integrated nested Laplace approximations (INLA-GP). We found QMC and MLMC to offer substantial computational savings when parameter sets are large and correlated and when the EVPPI is large. We also found that GP and INLA-GP were biased in those situations, whereas GAM cannot estimate EVPPI for large parameter sets.

Cost-effectiveness analysis is used to compare the costs and benefits of medical interventions, which are often combined as a net monetary benefit.^1^ Such analyses are internationally adopted by decision makers and health technology assessment agencies, including the National Institute for Health and Care Excellence (NICE) in the United Kingdom, the Institute for Clinical Effectiveness Review in the United States, the Canadian Agency for Drugs and Technology in Health in Canada, and the Pharmaceutical Benefits Advisory Committee in Australia. Costs and effects of interventions can be estimated using trial-based analysis or extrapolated over patient lifetimes using model-based decision analysis.^2^ Examples of decision models include decision trees, cohort Markov models, and individual patient microsimulations.^3^ These models estimate the net benefit of interventions as a function of input parameters such as treatment effectiveness, drug costs, or quality of life following an intervention. This function will be referred to as the net benefit (NB) function. Because the model parameters are estimated from data, we are uncertain about the true parameter values (due to imperfect information on them). This uncertainty in model parameters is propagated through the model to give our uncertainty in the true costs and effects. This in turn leads to a quantification of our uncertainty around the optimal treatment recommendation, which is known as decision uncertainty arising from imperfect information on input parameters. The expected value of perfect information (EVPI) is the expected improvement in decision making, often valued on the monetary scale, from gaining perfect information on all parameters.^4,5^ The expected value of partial perfect information (EVPPI) is the value of gaining perfect information on a subset of the parameters.^6^ These quantities have the potential to guide research funding, as studies costing more than the EVPI and EVPPI will not be cost-effective, but those costing less may be cost-effective, and this can be explored using the expected value of sample information (EVSI). The EVPPI is also a useful sensitivity analysis, as it can highlight parameters to which the decision is most sensitive.

Estimating PPI requires nesting one Monte Carlo simulation over the subset of parameters on which further research is being considered within a second Monte Carlo simulation of the remaining parameters. This procedure is termed *nested Monte Carlo* and requires evaluation of the NB function for all samples. EVPPI calculations can be computationally intensive, especially for economic models that involve individual-level simulation or Markov models with large numbers of states, and incur significant computational cost to evaluate the NB functions. As a result, standard nested Monte Carlo simulation often fails because it requires an impractical number of samples to obtain a reasonably precise estimate. In addition, estimation based on the standard nested Monte Carlo method is biased.^6,7^ Estimation of this bias is computationally expensive, as it requires comparison of EVPPI estimates based on different numbers of samples.^6,8^ Much recent effort has aimed to reduce the computational burden of EVPPI^7^ through approximating the conditional expected NB functions and thus replacing one of the Monte Carlo samples from nested Monte Carlo, by some functions that are less computationally intensive to evaluate. Success has been found through linear approximations to the conditional expected NB functions or exact algebraic solutions of the EVPPI, but these methods are specific to each model design and are not always appropriate, particularly for highly complex and nonlinear model structures.^9^ Meta-modeling through generalized additive models (GAMs), Gaussian processes, and integrated nested Laplace approximations (INLA-GP) is an elegant and general approach to reducing the computational burden of EVPPI.^10–12^ Gaussian process (GP) and GAM methods fit a regression model of NB on the input parameters to then estimate the conditional expected NB, thus removing the need for nested simulation. The INLA-GP method fits a 2-dimensional Gaussian process to a dimension-reduced sample of the model parameters and model outputs. These have been implemented in user-friendly online tools and software packages.^13,14^ However, all of these methods are based on approximating the conditional expected NB function, incurring a bias that is difficult to quantify. A prohibitively large number of samples may also be required to determine a sufficiently well-fitting regression function.

To fully capture decision uncertainty, cost-effectiveness decision models should reflect all the available relevant evidence. For model parameters in which there are multiple evidence sources available, evidence synthesis methods are used to pool the results, often using Bayesian inference evaluated using Monte Carlo Markov chain simulation.^1^ Relative treatment effects, for example, are commonly estimated using Bayesian network meta-analysis (NMA), which delivers a joint distribution for multiple treatment effects that are not available in closed form but instead represented by samples from an Markov chain Monte Carlo (MCMC) simulation. This poses 2 challenges for EVPPI calculation. First, it may require a very large number of samples to characterize the posterior distribution, for example, if correlations between parameters impede mixing of the sampler. In addition to needing a large number of samples, it can also be computationally expensive to generate each sample from the posterior, as for the NMA used in the directly acting oral anticoagulants (DOACs) for prevention of stroke in an atrial fibrillation Markov model.^15–18^ This can make nested Monte Carlo, or the generation of a sufficient number of samples to determine the regression function for GAM or GP methods, impractical. A further challenge with MCMC is that it is difficult, particularly using off-the-shelf general purpose Gibbs samplers, such as OpenBUGS,^19^ to generate the conditional distributions needed for EVPPI estimation by standard nested Monte Carlo. This motivates us to explore new computational methods for EVPPI.

In this article, instead of approximating the conditional expected NB function, we introduce 2 different Monte Carlo methods to reduce the number of samples and NB function evaluations needed for the same accuracy as the standard nested Monte Carlo method: multilevel Monte Carlo (MLMC) and quasi Monte Carlo (QMC). These Monte Carlo methods are unrelated to each other, but we explore them in parallel.

The MLMC method, introduced by Giles,^20,21^ has been successfully applied to many research fields, such as financial mathematics, mathematical biology, and uncertainty quantification. The first key idea of MLMC is to create a series of estimators for the quantity of interest, such as EVPPI, which are increasing in accuracy and increasing in computational cost. The first term, which is the lowest level, is the least accurate and computationally least intensive, whereas the last term, or highest level, is the most accurate and most expensive. All these terms have similar variance. By careful construction, consecutive estimators in the sequence are designed to be highly correlated. This gives the second key idea, which is that the differences between consecutive terms have much lower variance than the individual terms and thus require fewer total samples to estimate. A combined estimator for the quantity of interest can then be formed by adding the lowest-level estimator to a sum of the differences of consecutive terms, which is a sum over the levels of MLMC and has the highest accuracy. As this is formed of terms that have much lower variance, the total number of samples needed for estimation can be much reduced from that needed for standard (i.e., non-multilevel) nested Monte Carlo.^21^ Goda and others have used MLMC to construct an estimator of the EVPPI that has lower cost than standard nested Monte Carlo.^22,23^ Technical details of MLMC for EVPPI are provided in the “MLMC Estimation of EVPPI” section of this article. MLMC for EVPPI offers the greatest computational savings over Monte Carlo when there are correlations between model parameters. This is because greater correlation requires more inner samples and thus deeper levels of MLMC, which leads to proportionately greater computational savings over Monte Carlo. An additional benefit of MLMC for EVPPI is that it can be used to estimate the bias of EVPPI, and indeed EVPI, and this estimate can be used to form an unbiased estimator of EVPPI.^22,24^ Inference on EVPPI often focusses on the point estimate and disregards uncertainty in the estimate, which could have an impact on trial-funding decisions. An estimate of both the accuracy and precision of the estimator would therefore be useful, beyond the need to form an unbiased estimator. Previous work has applied MLMC for EVPPI to only simple models and has not considered the case in which the model input parameters have been estimated using Bayesian methods, and uncertainty in the parameters is represented by MCMC simulations rather than a closed-form distribution.

Standard Monte Carlo simulation uses pseudo–random-number generators that attempt to mimic truly random sequences.^25^ However, with truly random sequences, the generated sequences tend to include clusters of points separated by large gaps. An example is shown in Figure 1c, which is a standard Monte Carlo sample from a bivariate uniform distribution. These clusters and gaps tend to reduce the efficiency of estimators based on the sample. QMC^26^ is an alternative to standard Monte Carlo simulation that instead of using pseudo–random-number generators uses quasi-random sequences that have been specially designed to avoid bunching and gaps in the sampling space (known as “low-discrepancy” sequences). This results in an estimator with a lower variance, which in turn reduces the number of necessary samples and computational cost. However, QMC has not yet been applied to EVPPI.

**Figure 1:**
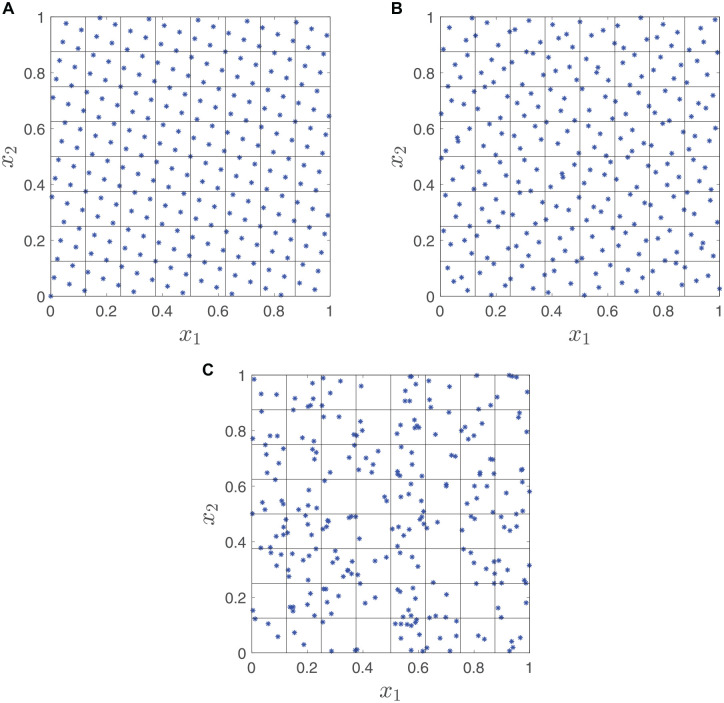
Generating points: quasis Monte Carlo versus Monte Carlo: (a) rank-1 lattice rule, (b) Sobol points, (c) pseudo-random points.

Both MLMC and QMC are variance-reduction methods that allow accurate estimation of the expectations with fewer samples than nested Monte Carlo does. Many other variance-reduction methods are available but out of the scope of this article (e.g., control variables, importance sampling, stratified sampling, and Latin hypercube; see Glasserman^27^ for more comprehensive description of these methods).

This article develops a QMC approach and extends an existing MLMC approach to efficient EVPPI estimation. In the “Methods” section, we begin with a discussion of the properties of Monte Carlo nested simulation for EVPI and EVPPI. How MLMC and QMC can reduce the number of samples needed and the extension of MLMC and QMC to MCMC samples are discussed in the “Applications” section. The “Results” section presnts an application to an artificial, but realistic, decision tree depression model with MCMC input parameters. The “Discussion” section presents an application to the published and highly complex DOACs for prevention of stroke in an atrial fibrillation Markov model.^16–18^ Further discussions and conclusions are provided in the “Conclusion” section. In the appendix, in addition to detailed experiment results, we also show how MLMC and QMC can be applied to value-of-information analysis, through a step-by-step explanation of the method and the provision of code examples.

## Methods

### Standard Nested Monte Carlo Estimation of EVPI and EVPPI

Suppose our model is a function of input parameters Z , and the NB in monetary units for each decision option *d* is given by $f_{d}$ . Then, assuming the decision maker is risk neutral and rational, the optimal decision option is that which is the expected net monetary benefit:

$$\underset{d\in D}{\max}E_{Z}[f_{d}(Z)],$$

where $E_{Z}$ is the expectation with respect to Z . If all uncertainty is eliminated in the parameter inputs, then we can choose the best decision and obtain monetary benefit $\underset{d\in D}{\max}f_{d}(Z)$ . Taking an expectation over the possible realizations of Z gives the expected monetary value based on perfect information

$$E_{Z}[\underset{d\in D}{\max}f_{d}(Z)].$$

Then, we define EVPI as the extra monetary value expected from learning the true value of Z :

(2.1) $$\mathrm{EVPI}=E_{Z}[\underset{d\in D}{\max}\;f_{d}(Z)]-\underset{d\in D}{\max}E_{Z}[f_{d}(Z)],$$

which indicates to the decision maker whether the optimal decision option is sensitive to the uncertainty in the model inputs and whether it is potentially cost-effective to fund new research on Z.

It may be that we are interested only in eliminating uncertainty on a subset X of model input parameters, and the remaining subset Y of parameters is still uncertain, where Z=(X,Y) . Then, given perfect information on X , the optimal decision option is that which maximizes the conditional expected net monetary benefit, where the expectation is over Y conditional on X ,

(2.2) $$\underset{d\in D}{\max}E_{Y|X}[f_{d}(X,Y)].$$

Taking an expectation over the possible realizations of X gives the expected monetary value

(2.3) $$E_{X}[\underset{d\in D}{\max}E_{Y|X}[f_{d}(X,Y)]],$$

Similarly, we define the EVPPI as the increased value expected from gaining perfect information about X :

(2.4) $$\mathrm{EVPPI}=E_{X}[\underset{d\in D}{\max}E_{Y|X}[f_{d}(X,Y)]]-\underset{d\in D}{\max}E_{Z}[f_{d}(Z)].$$

In most practical cases, there is no closed-form solution to the EVPI and EVPPI, and we must turn to numerical methods. One straightforward way is the nested Monte Carlo method, which approximates the expectation as an average of samples. Assuming we can sample from the distribution of Z , we can generate N independent samples $Z^{\left(1\right)},Z^{\left(2\right)},\ldots ,Z^{\left(N\right)}$ and approximate the EVPI by

(2.5) $$\hat{\mathrm{EVPI}}=\frac{1}{N}\sum_{n=1}^{N}\underset{d\in D}{\max}f_{d}(Z^{\left(n\right)})-\underset{d\in D}{\max}\frac{1}{N}\sum_{n=1}^{N}f_{d}(Z^{\left(n\right)}),$$

which is biased downward because of the positive bias of the second term.^8^

We can form a similar approximation for EVPPI if we can sample from X, and from Y given X. We first generate N samples $X^{\left(1\right)},X^{\left(2\right)},\ldots ,X^{\left(N\right)}$ , and for each $X^{\left(n\right)}$ , we then generate M samples of $Y^{\left(n,m\right)}$ according to the conditional distribution based on $X^{\left(n\right)}$ . Finally, we approximate the EVPPI by

(2.6) $$\begin{array}{c} \hat{\mathrm{EVPPI}}=\frac{1}{N}\sum_{n=1}^{N}\underset{d\in D}{\max}\frac{1}{M}\sum_{m=1}^{M}f_{d}(X^{\left(n\right)},Y^{\left(n,m\right)})\\ -\underset{d\in D}{\max}\frac{1}{\mathrm{NM}}\sum_{n=1}^{N}\sum_{m=1}^{M}f_{d}(X^{\left(n\right)},Y^{\left(n,m\right)}). \end{array}$$

The N samples $X^{\left(n\right)}$ are referred to as the “outer” samples, whereas the M samples of $Y^{\left(n,m\right)}$ , the summation over which is nested inside the summation over the outer samples, are referred to as the “inner” samples. Due to Jensen’s inequality, both terms in estimator (2.6) have positive bias. The estimator is therefore biased, but it is difficult to conclude whether the estimator is biased upward or downward.^8^

Both the bias and variance of the estimator are important. Bias relates to the accuracy of the estimate, whereas the variance relates to how precise the estimate is. A precise biased estimate can be very misleading, because it gives confidence but in the wrong thing. It is therefore good practice to report both, and as noted, the bias estimate can help obtain an unbiased estimate. In general, we want to find minimum variance unbiased estimators if possible. Therefore, we consider the mean square error (MSE) of the EVPPI, which is defined as

$$\mathrm{MSE}=E[|\hat{\mathrm{EVPPI}}-\mathrm{EVPPI}{|}^{2}],$$

and can be decomposed into two parts,

$$\mathrm{MSE}=(E[\hat{\mathrm{EVPPI}}-\mathrm{EVPPI}{])}^{2}+\mathrm{Var}[\hat{\mathrm{EVPPI}}],$$

that is, this is the square of the bias plus the variance of the estimator. For all numerical methods, a prescribed MSE $\varepsilon^{2}$ can be achieved by bounding the bias by ε/2 and the variance by $3\varepsilon^{2}/4$ , since $\mathrm{MSE}=\mathrm{Bia}s^{2}+\mathrm{Var}=(\varepsilon /2{)}^{2}+3\varepsilon^{2}/4=\varepsilon^{2}$ ; this choice is somewhat arbitrary, but we found in numerical experiments that the split has little impact on results. For a square root mean squared error (RMSE) ε , the total number of samples required by the standard nested Monte Carlo method is of the order of $\varepsilon^{-3}$ .^21^ This is because the number of outer samples to bound the variance is the inverse of the desired variance (i.e., the order of $\varepsilon^{-2}$ ), and the number of inner samples, per outer sample, to bound the bias is the inverse of the desired bias (i.e., the order of $\varepsilon^{-1}$ ). As both the variance and bias must be bounded, this gives the total order of inverse cube of the desired RMSE (i.e., $\varepsilon^{-3}$ ).

However, in practice, nested Monte Carlo is computationally expensive. In the following 2 sections, we introduce 2 advanced Monte Carlo methods to improve the computational efficiency and determine an appropriate number of samples to achieve a prescribed bias and obtain corresponding credible intervals systematically.

### MLMC Estimation of EVPPI

We follow the recently published approach of Giles and Goda^23^ to apply MLMC to the estimation of EVPPI. As explained in the introduction, the first key idea of MLMC is to create a series of estimators of the quantity of interest, in our case EVPPI, which are increasing in accuracy and increasing in computational cost. These estimators are carefully constructed to ensure that consecutive terms are correlated; this gives the second key idea that differences between consecutive terms have low variance and therefore require fewer total samples to estimate as compared with standard Monte Carlo. To illustrate how the MLMC method works, we first consider a simple example; full details on how to construct the necessary estimators for EVPPI will follow below.

First, consider 2 crude estimators for EVPPI, labeled ${\hat{e}}_{0}(N)$ and ${\hat{e}}_{1}(N)$ , each defined as the estimator (2.6) based on N outer samples of X but with M=1 and M=2 inner samples of Y , respectively, and each rewritten in the form of an average,

$${\hat{e}}_{0}(N)=\frac{1}{N}\sum_{n=1}^{N}e_{0}^{\left(n\right)},\;\;{\hat{e}}_{1}(N)=\frac{1}{N}\sum_{n=1}^{N}e_{1}^{\left(n\right)}$$

where

$$\begin{array}{c} e_{0}^{\left(n\right)}=\underset{d\in D}{\max}f_{d}(X^{\left(n\right)},Y^{\left(n,1\right)})-\underset{d\in D}{\max}\frac{1}{N}\sum_{i=1}^{N}f_{d}(X^{\left(i\right)},Y^{\left(i,1\right)})\\ e_{1}^{\left(n\right)}=\underset{d\in D}{\max}\frac{1}{2}(f_{d}(X^{\left(n\right)},Y^{\left(n,1\right)})+f_{d}(X^{\left(n\right)},Y^{\left(n,2\right)}))-\\ \;\underset{d\in D}{\max}\frac{1}{2N}\sum_{i=1}^{N}(f_{d}(X^{\left(i\right)},Y^{\left(i,1\right)})+f_{d}(X^{\left(i\right)},Y^{\left(i,2\right)})) \end{array}$$

${\hat{e}}_{0}(N)$ is more biased (essentially, it is the estimator [2.5] for EVPI) but requires half as many samples of Y to achieve the same precision as the less biased estimator ${\hat{e}}_{1}(N)$ . We can then construct a new estimator

$${\hat{e}}_{0}^{*}(N_{0},N_{1})={\hat{e}}_{0}(N_{0})+{\hat{d}}_{1}(N_{1})$$

with the same degree of bias as ${\hat{e}}_{1}(N)$ , by adding an estimator of the bias reduction, ${\hat{d}}_{1}$ , to the original biased estimator ${\hat{e}}_{0}$ . The bias reduction estimator is defined as the difference

$${\hat{d}}_{1}(N_{1})=\frac{1}{N_{1}}\sum_{n=1}^{N_{1}}\;d_{1}^{\left(n\right)}=\frac{1}{N_{1}}\sum_{n=1}^{N_{1}}(e_{1}^{\left(n\right)}-e_{0}^{\left(n\right)})$$

Then, if each term $d_{1}^{\left(n\right)}$ is calculated from a single sample $Y^{\left(n,1\right)}$ , rather than using a pair of different samples of $Y^{\left(n,1\right)}$ for $e_{1}^{\left(n\right)}$ and $e_{0}^{\left(n\right)}$ , and using a single sample of $X^{\left(n\right)}$ , the variance of ${\hat{d}}_{1}(N_{1})$ is lower. Consequently, half the number of samples are required for the new bias-reduced EVPPI estimator ${\hat{e}}_{0}^{*}$ to achieve a variance similar to the original less-biased estimator ${\hat{e}}_{1}$ . The sizes $N_{0},N_{1}$ of the samples used to obtain each term in the new estimator can be tuned to achieve the desired balance of variance and computational cost.

**Figure 2:**
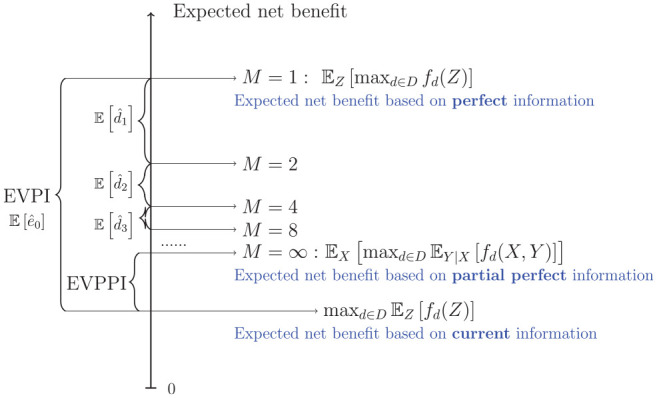
Illustration of multilevel Monte Carlo (MLMC) estimation of expected value of partial perfect information (EVPPI). The horizontal lines represent estimates of the expected net benefit under partial perfect information, using ℓ levels and $M=2^{\ell }$ inner samples. The MLMC estimate of EVPPI is the difference between the level ℓ estimate and the expected net benefit under current information. For ℓ=0 , this is the EVPI, whereas it converges to the true EVPPI as ℓ→∞ or, equivalently, as an increasing number of bias reduction terms $E\left[{\hat{d}}_{\ell }\right]$ are added to the ℓ=0 estimator.

MLMC is a natural extension of this principle. A sequence of estimators ${\hat{e}}_{0}(N_{0}),{\hat{e}}_{1}(N_{1}),{\hat{e}}_{2}(N_{2}),\ldots$ is constructed, with decreasing bias but also with increasing computational cost for the same precision. The ℓ th term in the sequence ${\hat{e}}_{\ell }(N_{l})$ is again defined by the standard Monte Carlo estimator (2.6), with $N_{\ell }$ outer samples and $M=2^{\ell }$ inner samples. Each level can have a different number of outer samples $N_{\ell }$ , but in practice, we use the same number N for each. The MLMC estimator of EVPPI is then constructed by starting with the most-biased estimator and adding a sequence of bias-reduction terms,

(2.7) $${\hat{e}}_{\ell }^{*}(N_{0},N_{1},\ldots ,N_{L})={\hat{e}}_{0}(N_{0})+\sum_{\ell =1}^{L}{\hat{d}}_{\ell }(N_{\ell })$$

where the ℓ th bias reduction term is ${\hat{d}}_{\ell }(N_{\ell })=\frac{1}{N_{\ell }}\sum_{n=1}^{N_{\ell }}(e_{\ell }^{\left(n\right)}-e_{\ell -1}^{\left(n\right)})$ , and $e_{\ell }^{\left(n\right)}$ is defined as before, so that the standard (non-MLMC) Monte Carlo estimator can be expressed as an average ${\hat{e}}_{\ell }(N_{\ell })=\frac{1}{N_{\ell }}\sum_{n=1}^{N_{\ell }}e_{\ell }^{\left(n\right)}$ (as illustrated in Figure 2). This MLMC EVPPI estimator has the same expectation (or degree of bias) as the most expensive of the Monte Carlo estimators in this sequence, ${\hat{e}}_{L}(N_{L})$ . Again, if the pair of components $e_{\ell }^{\left(n\right)},e_{\ell -1}^{\left(n\right)}$ in each term of the bias reduction estimator are evaluated using the same sample Y , then we will require half the number of samples to achieve the same precision as an estimator built from 2 independent samples of Y . The computational savings, compared with full Monte Carlo, accumulate as the bias reduction terms are added. The consequence is that the MLMC EVPPI estimator requires fewer samples to achieve the same degree of precision as the Monte Carlo estimator ${\hat{e}}_{L}(N_{L})$ , without affecting the bias.

As shown in Giles and Goda,^23^ given a required RMSE of ε , the total number of samples required is of an order of magnitude of $\varepsilon^{-3}$ for the standard Monte Carlo method, but $\varepsilon^{-2}$ for the MLMC method at best, as long as the number of inner samples for ${\hat{e}}_{\ell }(N_{\ell })$ is set to $2^{\ell }$ . As L is increased, the bias of the resulting EVPPI estimator reduces, so that it converges to the true EVPPI with diminishing returns (as illustrated in Figure 2).

The number of levels L , and the number of outer samples $N_{\ell }$ in each level, can be tuned so that the MLMC estimator achieves a specific balance of bias, precision, and computational speed, as outlined in the “Estimating Number of Levels *L* and Samples *N_l_* Required for MLMC” in the appendix, along with 2 other refinements to improve performance of the algorithm.

### QMC Estimation of EVPPI

As explained in the introduction, standard Monte Carlo samples are pseudo-random (i.e., random samples from a distribution of interest) that are generated deterministically by a computer but statistically indistinguishable from truly random samples. Quasi-random samples, on the other hand, although not statistically random, can be used to approximate the distribution of interest, with fewer samples required for the same level of precision. Some examples of quasi-random samples are shown in Figure 1a and b, for a bivariate uniform distribution. These can be seen to cover the sampling space more evenly than a standard Monte Carlo sample (Figure 1c).

For example, given a scalar random variable X whose cumulative distribution function Φ is known, we can generate random samples $X^{\left(n\right)}$ of X by sampling $U^{\left(n\right)}$ from a standard uniform distribution and setting $X^{\left(n\right)}=\Phi^{-1}(U^{\left(n\right)})$ . Then we can estimate, for example, E[X] , from a sample of size n by $\sum_{n=1}^{N}X^{\left(n\right)}/N$ . In standard Monte Carlo, $U^{\left(n\right)}$ are chosen randomly. In QMC, we generate low-discrepancy sequences, which are points distributed over [0,1] more evenly. This approach generalizes to s -dimensional random variables X , by generating $U^{\left(n\right)}$ distributed uniformly over the $[0,1{]}^{s}$ hypercube (after applying a Cholesky decomposition if the X are correlated, see, e.g., Briggs et al.^3^).

Two common approaches for generating low-discrepancy sequences are rank-1 lattice rules (illustrated in Figure 1a) and Sobol sequences (Figure 1b).

- Rank-1 lattice rules generate samples as ( $U^{\left(n\right)}=\frac{n}{N}z\mathrm{mod}1$ ), where z is an s -dimensional vector whose components are integers that share no positive integer divisors, other than 1, with the sample size N . The fraction $\frac{n}{N}$ is multiplied by each element of z , and the mod1 operation keeps only the fractional part of $\frac{n}{N}z$ . For example, if X consists of 1 scalar parameter, this would produce an evenly spaced sequence on [0,1], equivalent to equally spaced quantiles of the distribution of X . For 2 (or more) random variables, this produces an evenly spaced “rotated” grid of points, which will capture their correlation better than a simple grid (Figure 1a). Methods for constructing such a vector are explained by Hickernell.^28^
- Sobol sequences $U^{\left(n\right)}$ have the property that for small dimensions s<40 , the subsequence $2^{m}\leq n\leq 2^{m+1}$ of length $2^{m}+1$ , for any m≥s , has precisely $2^{m-s}$ points in each of the cubes of volume $2^{-s}$ formed by bisecting the unit hypercube in each dimension. These can be constructed as explained by Owen.^29^ For example, cutting it into halves in any dimension, each has $2^{m-1}$ points; cutting it into quarters in any dimension, each has $2^{m-2}$ points. We chose to use Sobol sequences as they are generated easily using the R package “randtoolbox,” illsutrated in our supplementary code.^30^

In the bivariate uniform example, using a rank-1 lattice method (Figure 1a), there are exactly 4 points in each small square, and using a Sobol sequence (Figure 1b), there are roughly 3 to 5 points in each small square. Thus, the sample space is covered more evenly than in the Monte Carlo method (Figure 1c).

A QMC estimator of EVPPI is defined by substituting the QMC sample $\Phi^{-1}(U^{\left(n\right)})$ for the standard Monte Carlo sample $X^{\left(n\right)}$ in equation (2.6). However, as the QMC sample is deterministic, it is difficult to quantify uncertainty in the estimate arising from the limited sample size. To obtain a credible interval, we instead use randomised QMC. This procedure generates K sets of QMC points $\{U^{\left(k,n\right)}{\}}_{1\leq n\leq N}$ for k=1,2,…,K as follows, where a choice of 8 to 32 for K has been empirically demonstrated as sufficient.^31^ We do not want to use a too large K , as the larger K is, the more precision we have to sacrifice to obtain the credible interval.

To do this for Sobol sequences, we perform digital scrambling using a bitwise exclusive-or operation. It maintains the low discrepancy by implementing a uniform random permutation of 0 and 1 for the binary expression of each sample $U^{\left(k,n\right)}$ .^29^ This is also implemented in the “randtoolbox” R package and illsutrated in our supplementary code.^30^

Then, the randomized QMC estimator of EVPPI is defined by substituting $\Phi^{-1}(U^{\left(k,n\right)})$ for the standard Monte Carlo sample $X^{\left(n\right)}$ and then averaging over random samples k

(2.8) $$\frac{1}{K}\sum_{k=1}^{K}\frac{1}{N}\sum_{n=1}^{N}g(\Phi^{-1}(U^{\left(k,n\right)})).$$

For the estimation of EVPPI, in most practical cases, the random variables X have standard distributions whose inverse cumulative distribution functions $\Phi^{-1}$ can be computed easily. The exception is where the joint distribution for Z is not known in closed form but instead represented by samples from an MCMC simulation. This topic is discussed in detail in the appendix.

When using QMC for EVPPI, we apply it only to the outer samples X , because, in practice, we do not need a great number of inner samples Y to achieve acceptable accuracy, that is of order of $\varepsilon^{-1}$ samples as explained in the “Standard Nested Monte Carlo Estimation of EVPI and EVPPI” section. Simulating larger numbers of inner samples will not be of benefit, as it would give a very accurate approximation only for each fixed outer sample, and the variance of the outer sample continues to have a larger impact on the total error.

For QMC to achieve an MSE $\varepsilon^{2}$ , we still need the number of inner samples per outer sample to be an order of magnitude of $\varepsilon^{-1}$ , but only need about $\varepsilon^{-1}$ outer samples, because of the properties of low-discrepancy sequences.^26^ Hence, the total computational cost is an order of about $\varepsilon^{-2}$ , compared with $\varepsilon^{-3}$ for standard Monte Carlo. This is about the same cost as MLMC, as we will show in the context of 2 real applications in the “Applications” section. Because low-discrepancy sequences reduce variance in nonnested Monte Carlo, QMC reduces the computational cost to estimate the total EVPI. In our numerical tests, we will include an assessment of this property.

However, similar to the standard MC method, QMC also fails to provide an estimate of bias. In the numerical tests of this article, we use MLMC only to estimate the bias and determine the number of inner samples required to bound the bias.

## Applications

We tested our methods on 2 applications. One is a simplified depression model, while the other is a real atrial fibrillation model comparing DOACs for the prevention of stroke. The latter model, along with its results, is described in the appendix (section 7.4).

### Simplified Cost-Effectiveness Model in Depression

**Figure 3:**
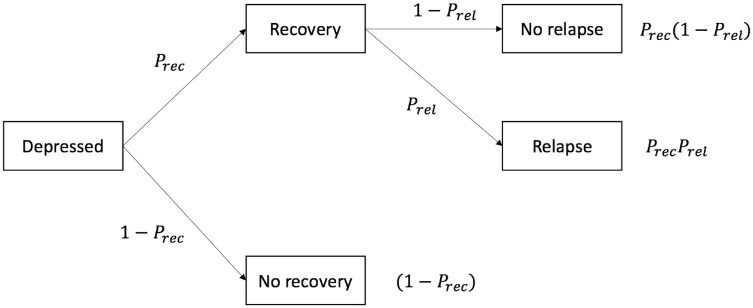
Decision tree for depression toy model (probabilities are defined in Table 2).

We applied our MLMC and QMC EVPPI estimators to an artificial model comparing options for the treatment of depression. We adopted the decision tree structure illustrated in Figure 3. This is based on a previously published model but is populated with artificial quality of life and costs of outcomes as well as a Bayesian NMA, implemented using MCMC, of response and relapse outcomes from constructed randomized controlled trial data.^32^ There are 3 options compared: no treatment, cognitive behavioral therapy (CBT), and antidepressants. We label these options d=1,2,3 respectively. The same structure is used for both treated (by any treatment) and untreated patients, but the probabilities of recovery and relapse depend on the treatment. In this model, patients begin their treatment (or no treatment) in the “Depressed” node and move to “Recovery” if they have an initial response to treatment and “No recovery” if not. Following “Recovery,” patients can either experience a relapse and end in the “Relapse” node or remain healthy in the “No relapse” node.

Costs and quality-adjusted life-years (QALYs) associated with 30 y (approximately lifetime) in the final states are assumed to follow normal distributions (Table 1).

**Table 1:** 30-y Costs and Quality-Adjusted Life-Years for the Depression Toy Model

	Recovery, No Relapse	Recovery, Relapse	No Recovery
Log cost	$C_{\mathrm{rec}}\sim N(1000,50^{2})$	$C_{\mathrm{rel}}\sim N(2000,100^{2})$	$C_{\mathrm{nrec}}\sim N(2500,125^{2})$
Quality-adjusted life-year	$Q_{\mathrm{rec}}\sim N(26,2^{2})$	$Q_{\mathrm{rel}}\sim N(23,3^{2})$	$Q_{\mathrm{nrec}}\sim N(20,4^{2})$

As given in Table 2, we assumed Beta distributions for the probabilities of relapse and recovery on no treatment. Log odds ratios of recovery and relapse on CBT and antidepressants come from 2 NMAs, each of which consist of 5 trials based on constructed data. These data were analyzed using a Bayesian binomial outcomes logistic link NMA implemented in the OpenBUGS software version 3.2.3 rev 1012,^19,33^ which generated MCMC samples of the posterior distributions for the log odds of relapse and recovery. Because conditional distributions are necessary for EVPPI, we used the multivariate normal distributions to produce the following approximate posterior distributions.

$$\left[\begin{array}{c} {\mathrm{lor}}_{\mathrm{rec}}^{\left(2\right)}\\ {\mathrm{lor}}_{\mathrm{rec}}^{\left(3\right)} \end{array}\right]\sim N\left(\left[\begin{array}{c} 0.99\\ 1.33 \end{array}\right],\left[\begin{array}{cc} 0.22 & 0.15\\ 0.15 & 0.20 \end{array}\right]\right),\left[\begin{array}{c} {\mathrm{lor}}_{\mathrm{rel}}^{\left(2\right)}\\ {\mathrm{lor}}_{\mathrm{rel}}^{\left(3\right)} \end{array}\right]\sim N\left(\left[\begin{array}{c} -1.48\\ -0.40 \end{array}\right],\left[\begin{array}{cc} 0.14 & 0.05\\ 0.05 & 0.11 \end{array}\right]\right).$$

The NB for treatment option d for a patient in our model is

(3.1) $$\begin{array}{c} NB^{\left(d\right)}=\lambda [P_{\mathrm{rec}}^{\left(d\right)}(1-P_{\mathrm{rel}}^{\left(d\right)})Q_{\mathrm{rec}}+P_{\mathrm{rec}}^{\left(d\right)}P_{\mathrm{rel}}^{\left(d\right)}Q_{\mathrm{rel}}+(1-P_{\mathrm{rec}}^{\left(d\right)})Q_{\mathrm{nrec}}]\\ \;-[P_{\mathrm{rec}}^{\left(d\right)}(1-P_{\mathrm{rel}}^{\left(d\right)})C_{\mathrm{rec}}+P_{\mathrm{rec}}^{\left(d\right)}P_{\mathrm{rel}}^{\left(d\right)}C_{\mathrm{rel}}+(1-P_{\mathrm{rec}}^{\left(d\right)})C_{\mathrm{nrec}}+C_{\mathrm{treat}}^{\left(d\right)}], \end{array}$$

where $C_{\mathrm{treat}}=(0,300,30)$ is the fixed initial treatment cost.

In our case, $f_{d}(Z)=NB^{\left(d\right)},$ the NB of treatment d given

$$Z=(P_{\mathrm{rec}}^{\left(d\right)},P_{\mathrm{rel}}^{\left(d\right)},C_{\mathrm{rec}},C_{\mathrm{rel}},C_{\mathrm{nrel}},Q_{\mathrm{rec}},Q_{\mathrm{rel}},Q_{\mathrm{nrel}},{\mathrm{lor}}_{\mathrm{rec}}^{\left(2\right)},\;{\mathrm{lor}}_{\mathrm{rel}}^{\left(2\right)},{\mathrm{lor}}_{\mathrm{rec}}^{\left(3\right)},\;{\mathrm{lor}}_{\mathrm{rel}}^{\left(3\right)}).$$

We estimate the EVPPI for 4 subsets X of Z. These are the probabilities of recovery and relapse $X=(P_{\mathrm{rec}}^{\left(1\right)},\;P_{\mathrm{rel}}^{\left(1\right)},\;{\mathrm{lor}}_{\mathrm{rec}}^{\left(2\right)},\;{\mathrm{lor}}_{\mathrm{rel}}^{\left(2\right)},{\mathrm{lor}}_{\mathrm{rec}}^{\left(3\right)},\;{\mathrm{lor}}_{\mathrm{rel}}^{\left(3\right)}),$ the costs and QALYs $X=(C_{\mathrm{rec}},C_{\mathrm{rel}},C_{\mathrm{nrel}},Q_{\mathrm{rec}},Q_{\mathrm{rel}},Q_{\mathrm{nrel}}),$ the log odds ratios for CBT $X=({\mathrm{lor}}_{\mathrm{rec}}^{\left(2\right)},\;{\mathrm{lor}}_{\mathrm{rel}}^{\left(2\right)}),$ and the log odds ratios for antidepressants $X=({\mathrm{lor}}_{\mathrm{rec}}^{\left(3\right)},\;{\mathrm{lor}}_{\mathrm{rel}}^{\left(3\right)}).$

**Table 2:** Probabilities of Events for the Depression Toy Model^a^

d	Treatment	$P_{\mathrm{rec}}^{\left(d\right)}$	$P_{\mathrm{rel}}^{\left(d\right)}$
1	No treatment	Beta(6,200)	Beta(2,100)
2	Cognitive behavioral therapy	$\mathrm{expit}(\mathrm{logit}(P_{\mathrm{rec}}^{\left(1\right)})+{\mathrm{lor}}_{\mathrm{rec}}^{\left(2\right)})$	$\mathrm{expit}(\mathrm{logit}(P_{\mathrm{rel}}^{\left(1\right)})+{\mathrm{lor}}_{\mathrm{rel}}^{\left(2\right)})$
3	Antidepressant	$\mathrm{expit}(\mathrm{logit}(P_{\mathrm{rec}}^{\left(1\right)})+{\mathrm{lor}}_{\mathrm{rec}}^{\left(3\right)})$	$\mathrm{expit}(\mathrm{logit}(P_{\mathrm{rel}}^{\left(1\right)})+{\mathrm{lor}}_{\mathrm{rel}}^{\left(3\right)})$

a The expit is the inverse of the logistic link function with definition $\mathrm{expit}(x)=\frac{1}{\left(1+e^{-x}\right)}$ . “lor” is the log odds ratio.

## Results

### MLMC and QMC Results for Simplified Cost-Effectiveness Model in Depression

For comparison, we set the MSE to be 0.25 ( ε=0.5 ) by bounding the bias by 0.25 and variance by 0.1875 . Table 3 shows the number of samples needed as the computational cost to achieve a 0.25 MSE.

**Table 3:** Comparison of Computational Cost Measured in Units of $10^{6}$ Samples^a^

EVPPI	Estimates	95 % Credible Interval	Computational Cost		
			MC	MLMC	QMC
$\begin{array}{c} P_{\mathrm{rec}}^{\left(d\right)},P_{\mathrm{rel}}^{\left(d\right)},{\mathrm{lor}}_{\mathrm{rec}}^{\left(2\right)},{\mathrm{lor}}_{\mathrm{rel}}^{\left(2\right)}\\ {\mathrm{lor}}_{\mathrm{rec}}^{\left(3\right)},\;{\mathrm{lor}}_{\mathrm{rel}}^{\left(3\right)} \end{array}$	275	[274, 277]	15.14	26.93	5.63
Cost and QALYs	287	[286, 289]	13.02	22.85	5.12
$\mathrm{lo}r_{\mathrm{rec}}$ and $\mathrm{lo}r_{\mathrm{rel}}$ CBT	7	[6, 9]	787.20	109.90	8.19
$\mathrm{lo}r_{\mathrm{rec}}$ and $\mathrm{lo}r_{\mathrm{rel}}$ antidepressants	1	[0, 3]	78.90	61.86	6.23

a $P_{\mathrm{rec}}$ , probability of recovery; $P_{\mathrm{rel}}$ , probability of relapse following recovery; $\mathrm{lo}r_{\mathrm{rec}}$ , log odds ratios of recovery for CBT and antidepressants compared to no treatment; $\mathrm{lo}r_{\mathrm{rel}}$ , log odds ratios of relapse for CBT and antidepressants compared with no treatment; CBT, cognitive behavioral therapy; MC, standard nested Monte Carlo; MLMC, multilevel Monte Carlo; QALY, quality-adjusted life-year; QMC, quasi Monte Carlo.

From Table 3, we can see that QMC achieves the same degree of accuracy with lower computational cost than standard nested MC and MLMC when the optimal number of inner samples is determined, because QMC has the smallest numbers in the “Computational Cost” column in the table. MLMC starts to show computational savings only relative to standard nested MC for the calculation of EVPPI for $\mathrm{lo}r_{2}$ and $\mathrm{lo}r_{3},$ since these parameters are correlated. Further cost-effectiveness results are provided in appendix section 7.2.1.

To reproduce the complexity that may be encountered in a real cost-effectiveness model, we extended our example from 3 to 20 treatment options. The model structure, outcome costs, and outcome QALYs remained the same, but treatment effects and costs were randomly generated. Both MLMC and QMC continue to work well, and QMC still has the lowest computational costs given the number of inner samples. Full details of the model and EVPPI results are provided in appendix sections 7.2.2 and 7.2.3.

### Comparison with Regression Approximation Methods on a Simplified Cost-Effectiveness Model in Depression

We also calculated the EVPPI using the regression approaches of the GAM and GP, as implemented in the R code of Sheffield Accelerated Value of Information (SAVI) and stochastic partial differential equations INLA-GP, as implemented in the R package BCEA.^12,14,34^ Since the GP method requires large matrices to be inverted, SAVI is restricted to the first 7500 samples from an economic model for this method, while BCEA struggles with larger samples due to memory restrictions; also, the primary advantage of these methods is their ability to estimate EVPPI with fewer samples and thus lower computational cost. For these reasons, we used only 7500 samples for the comparison. To provide an approximately fair comparison, we restricted MC, QMC and MLMC to 50,000 samples, which has a similar computation time to running 7500 samples followed by GAM, GP, and INLA-GP regression. Note that SAVI automatically uses GP for parameter sets larger than 5, and in this example, with 6 parameters, GAM methods are computationally infeasible,^13^ although the BCEA implementation of GAM also suffers with memory restrictions when applied to larger parameter sets. As a consequence, only the GP-based methods are possible for estimating the EVPPI for the 2 sets of 6 parameters. The R code of SAVI provides the estimates of both standard error (i.e., the standard deviation of the estimator) and the upward bias.^13^ Similar to MLMC and QMC, the MSE of SAVI would be the sum of the variance of the estimator and the square of the upward bias. Furthermore, BCEA INLA-GP cannot estimate standard errors.

Table 4 suggests that both GP and GAM perform better for 2-dimensional sets of log odds ratios, giving estimates close to the reference and small standard errors. On the more complex 6-dimensional set of probabilities and the 6-dimensional cost and QALYs, QMC and MLMC are close to the reference MC estimate, but only QMC offers computational savings (lower RMSE) over standard MC. On these 6-dimensional sets, the point estimate for probabilities of GP is in agreement with those of MC methods, although the RMSE is larger and estimates appear to be biased. The point estimate for cost and QALYs of GP has a much smaller bias and standard error but is not consistent with the reference MC estimate. INLA-GP agrees on the 6-dimensional probabilities but gives a poor estimate of the EVPPI of the costs and QALYs. This was indicated by the BCEA diagnostic quantile-quantile plots (qq-plots), which suggested poor fit of the underlying regression model for INLA-GP (details in appendix section 7.3).

**Table 4:** Comparison of Estimates and Uncertainties for EVPPIs in the Depression Toy Model^a^

Parameter (Size)	MC Reference (RMSE) (Bias, SE)	MC (RMSE) (Bias, SE)	QMC (RMSE) (Bias, SE)	MLMC (RMSE) (Bias, SE)	GAM (RMSE) (Bias, SE)	GP (RMSE) (Bias, SE)	INLA-GP
Probabilities (6)	275 (0.5) (0.25, 0.43)	273.62 (5.19) (2.59, 4.48)	281.20 (3.03) (2.59, 1.57)	274.84 (12.00) (6.00, 10.38)	NA	322.19 (100.17) (89.66, 44.68)	293.44
Costs and QALYs (6)	287 (0.5) (0.25, 0.43)	286.86 (5.46) (2.73, 4.72)	286.93 (4.58) (2.73, 3.68)	285.13 (9.80) (4.90, 8.48)	NA	557.03 (0.77) (0.09, 0.76)	547.42
CBT (2)	7 (0.5) (0.25, 0.43)	29.53 (20.47) (9.72, 18.01)	44.65 (20.44) (9.72, 17.98)	22.03 (19.44) (9.72, 16.82)	12.26 (10.52) (5.52, 8.96)	11.91 (12.26) (1.74, 12.14)	13.83
Antidepressant (2)	1 (0.5) (0.25, 0.43)	0.28 (18.73) (8.00, 16.94)	5.12 (16.58) (8.00, 14.52)	4.10 (16.00) (8.00, 13.84)	1.56 (18.10) (14.20, 11.23)	6.62 (10.95) (4.87, 9.81)	4.81

CBT, cognitive behavioral therapy; GAM, generalized additive model; GP, Gaussian process; INLA-GP, integrated nested Laplace approximation; MC, Monte Carlo; NA, not applicable; QALY, quality-adjusted life-year; QMC, quasi Monte Carlo; RMSE, root mean squared error; SE, standard error.

a Use 50,000 samples for MC, MLMC, and QMC and 7500 samples for GAM, GP, and INLA-GP. The MC reference value is that from Table 3.

The EVPI and its RMSE for the depression model were 574.91 and 3.12 using standard MC, respectively, and 575.47 and 2.37 using QMC. The lower RMSE suggests computational savings from using QMC.

## Discussion

This article has developed more efficient Monte Carlo sampling methods to estimate EVPPI in complex and realistic cost-effectiveness models. We have generalized the previously published MLMC estimator for EVPPI to models in which the distributions of input parameters are known only through MCMC samples and demonstrated that it can be much more efficient than standard MC for computing many-parameter EVPPI in a realistically complex economic model. We have also provided the first implementation of QMC to EVPPI estimation. Unlike previous work on efficient EVPPI estimation via model regression and INLA-GP,^12,13,34^ we have separately quantified both bias and variance of our estimators and included them in our credible intervals. We have compared the accuracy of the QMC and MLMC methods relative to the GAM, GP, and INLA-GP regression approaches for the same approximate computational cost. The MLMC estimator can easily give an estimate of the bias, and this can be used to obtain credible intervals for MLMC but also for both QMC and standard nested MC estimators, so long as MLMC is conducted in addition to either QMC or MC.

The main contributions of this article have been to extend MLMC for EVPPI and develop QMC for EVPPI. Although our results suggest that QMC and MLMC can provide substantial computational savings over MC, and greater accuracy than regression techniques, we have explored only 2 example models. A more formal investigation would be needed to fully compare MC, MLMC, QMC, and regression. This could involve building a range of models with increasing numbers of health states, input parameters, and decision options and exploring higher correlation and greater MCMC on the input parameters. Further theoretical research is also required to understand why each of the methods performs well under different circumstances. Without such a program, it would be inappropriate to generalize and make firm recommendations. However, we make some observations based on our empirical findings and the theoretical understanding of QMC and MLMC.

Our depression example in the “Results” section and atrial fibrillation DOACs example in appendix section 7.4 may indicate a general trend that QMC outperforms MLMC when the underlying model is simple (e.g., decision tree or Markov model with few states, few parameters, low correlation) and MLMC outperforms QMC when the model is complex (e.g., Markov model with many states, large numbers of correlated or MCMC parameters). Our depression toy example also indicated that MC can outperform MLMC when the models are simple. If an estimate of the bias is required, MLMC must be employed as Monte Carlo and QMC, and regression cannot estimate the bias. Furthermore, if very high accuracy, or low bias, is required, MLMC is likely best, as higher levels of MLMC will eventually achieve any accuracy; however, this may not be computationally feasible. Conversely, if the EVPPI is very small, which could be found by an initial run of Monte Carlowith few samples, then MLMC may offer limited computational savings over Monte Carlo. Indeed, we found in the depression toy example that MLMC could not provide an estimate for small EVPPI in a reasonable time. Theoretically, QMC should be no worse than Monte Carlo in all cases. However, the computational savings depends on the specific case. In practice, furthermore, QMC can perform worse than Monte Carlo, as was seen for the simple trial example in the DOACs model, where our implementation failed to produce an estimate in a reasonable time as too many inverse distribution function evaluations were needed.

We have also found that MLMC and QMC provide more reliable and computationally efficient estimates of the EVPPI than regression techniques when parameter sets are large and when the EVPPI value is large and a precise EVPPI estimate is required. Conversely, and in line with MLMC theory, we did not find a huge advantage over standard Monte Carlo or approximation methods when the EVPPI or parameter sets are small. We also do not expect an advantage of MLMC or QMC when applied to single-parameter EVPPI. However, we found using QMC conferred computational savings over standard Monte Carlo when estimating the total EVPI.

From our experiment, to incorporate inputs whose distribution is estimated by MCMC, our MLMC and QMC methods do not rely on many approximations or distributional assumptions. Note that in QMC, we use Principle Component Analysis (PCA) to identify which of the first 2 dimensions of X to sort on. The reason we use 2 dimensions is that we find that, for this problem, the first 2 dimensions of PCA provide reasonably good approximation and are not computationally expensive. Still, it was necessary to assume an Multivariate Normal (MVN) approximation. This was required for sampling from conditional distributions in which parameters were correlated in both MLMC and QMC and to estimate the inverse cumulative distributions for QMC. We presented a method of resampling from the MCMC samples using random or quasi-random numbers, thus avoiding the need for large numbers of MCMC samples or the inverse cumulative distribution, although this does not address the need for correlated samples. The MVN approximation is likely suitable for parameters such as relative treatment effects expressed as log odds or hazard ratios.^33^ As an alternative, we explored multivariate t-distributions to capture possible fat tails.^35^ However, the credible interval largely coincided with what was provided by the MVN approximation (see appendix section 7.7for detailed results).

A primary disadvantage of the MLMC method from an applied perspective is the requirement for more than a single random sample from a standard probabilistic sensitivity analysis. The model regression approaches of GP, GAM, or INLA-GP require only samples from the input parameters and the estimated costs and QALYs to provide estimates of EVPPI. MLMC requires implementation of a more complicated form of nested simulation than in standard Monte Carlo, plus sampling from conditional distributions to provide the necessary estimates. This challenge may limit its applicability. We have provided R code in the appendix for both the depression and DOACs model, which users can adapt to their own models. QMC also requires conditional sampling but can use the same code as standard nested Monte Carlo; it requires only a switch to quasi-random numbers for the random-number generators (example code also provided in the appendix). However, we found that QMC did not work as well in the case of highly complex cost-effectiveness models such as Markov models.

Although we have used very large numbers of simulations ( $10^{5}$ to $10^{8}$ samples) for trial-funding decisions, such large numbers of samples and low RMSE may not be necessary. Such decisions are made by applying the population EVPI and EVPPI to the cost of a proposed trial. The population EVPI and EVPPI are the per-person values we have discussed but scaled to the annual number of patients who would benefit, scaled and discounted over the technology lifetime for which we expect the research to be relevant. In the DOACs example, if we assume 5000 patients per year, discounting at 1.035, and summing over a technology lifetime of 10 y gives a total population of approximately 43,038 patients. Scaling the value of a “simple trial” comparing apixaban to dabigatran estimated by only 50,000 MC samples from Table 4 gives a population EVPPI of £8.69 million and RMSE of £2.27 million. This implies lower and upper 95% credible bounds of about £4.25 million and £13.14 million, respectively. If a randomized controlled trial comparing these DOACs cost £3 million, it would be below the lower bound, and the decision would be to fund; no greater accuracy or lower variance would be needed. However, trial costs and EVPPI may be closer in other situations.

Our work so far has been limited to EVPPI, but to truly determine whether a future study will be cost-effective, we would need an estimate of the EVSI.^5^ Although the EVPPI for a set of parameters may be large, the EVSI for all but impractical study designs could be small. Despite its importance, EVSI is rarely estimated because of the unfamiliarity with the skills required and, for trials potentially informing many parameters, high computational requirements.^36^ Efficient sampling schemes, such as importance sampling, Gaussian approximation, and moment matching approaches have been explored, but a general solution applicable to all model and trial complexities remains elusive.^11,37,38^ Implementing QMC for EVSI would be straightforward, although the computational savings are unknown. Conversely, constructing an MLMC estimator, necessary for bias estimation, would require considerable research effort. However, MLMC and QMC may provide an accurate and efficient estimator of EVSI and improve its adoption by the heath economic community.

## Conclusion

In this article, we developed MLMC and QMC for the computation of EVPPIs and applied them a decision tree and Markov model example. In some cases, both methods improved the computational efficiency of the standard nested MC method, although they are more difficult to implement than standard MC. We found that for small numbers of parameters and small EVPPI values, GAM and GP were sufficient for EVPPI estimation. However, for large numbers of parameters and EVPPI values, where GAM is not feasible, MLMC and QMC can provide substantially more accurate and precise estimates than GP and INLA-GP. Further theoretical and empirical research is required to make formal recommendations between standard nested MC, QMC, MLMC, and the regression techniques.

## Supplemental Material

sj-bib-2-mdm-10.1177_0272989X211026305 – Supplemental material for Multilevel and Quasi Monte Carlo Methods for the Calculation of the Expected Value of Partial Perfect Information Click here for additional data file. Supplemental material, sj-bib-2-mdm-10.1177_0272989X211026305 for Multilevel and Quasi Monte Carlo Methods for the Calculation of the Expected Value of Partial Perfect Information by Wei Fang, Zhenru Wang, Michael B. Giles, Chris H. Jackson, Nicky J. Welton, Christophe Andrieu and Howard Thom in Medical Decision Making

sj-pdf-1-mdm-10.1177_0272989X211026305 – Supplemental material for Multilevel and Quasi Monte Carlo Methods for the Calculation of the Expected Value of Partial Perfect Information Click here for additional data file. Supplemental material, sj-pdf-1-mdm-10.1177_0272989X211026305 for Multilevel and Quasi Monte Carlo Methods for the Calculation of the Expected Value of Partial Perfect Information by Wei Fang, Zhenru Wang, Michael B. Giles, Chris H. Jackson, Nicky J. Welton, Christophe Andrieu and Howard Thom in Medical Decision Making
