# Supplementary material for: Multilevel and Quasi Monte Carlo Methods for the Calculation of the Expected Value of Partial Perfect Information
Source: Med Decis Making. 2021 Jul 7;42(2):168–81. doi: 10.1177/0272989X211026305 (PMC8777326; doi:10.1177/0272989X211026305)
Supplement: sj-pdf-1-mdm-10.1177_0272989X211026305 – Supplemental material for Multilevel and Quasi Monte Carlo Methods for the Calculation of the Expected Value of Partial Perfect Information [file sj-pdf-1-mdm-10.1177_0272989X211026305.pdf]

7 Appendix

7.1 Further details of MLMC

7.1.1 Estimating number of levels L and samples N_l required for MLMC

As mentioned, the number of levels L, and the number of outer samples N_ℓ in each level, can be tuned so that the MLMC estimator achieves a specific balance of bias, precision and computational speed. For example, we might want to minimise the cost to obtain a RMSE of at most ε and a bias of at most $\varepsilon/2$.

It was shown in [23] that the expectation and variance of the level ℓ estimators are approximately $C_1 2^{-\alpha\ell}$ and $C_2 2^{-\beta\ell}$. Therefore we can estimate $\log(C_1)$ and $-\alpha$ as the intercept and slope of a linear regression fitted to the three data points from levels $\ell = 0, 1, 2$ with $\log d_\ell$ as the outcome and ℓ as the predictor, similarly for $\log(C_2)$ and $-\beta$. It was shown in [23] for the standard MLMC method (Section 2.2) we have $\alpha = 1$ due to the central limit theorm, $\beta = 1$ due to the smoothness of the net benefit function f_d. Alternative sampling schemes where α and β are not 1 are possible, and discussed in later sections, and in these cases the procedure below can be used to estimate them by regression.

The step-by-step procedure is as follows:

1. Run a small pilot sample, for example of size $N_l = 1000$ for each ℓ and estimate the expectation and variance at the first three levels $\ell = 0, 1, 2$ (i.e. $\hat{e}_0, \hat{d}_1, \hat{d}_2$). At $\ell = 1$, for example, the expectation would be $\hat{d}_1(N_1) = \frac{1}{N_1}\sum_{n=1}^{N_1} d_1^{(n)}$ and the variance $\hat{V}_1(N_1) = \frac{1}{N_1}\sum_{n=1}^{N_1}\left(d_1^{(n)} - \hat{d}_1(N_1)\right)^2$.
2. Use regression on the three points to estimate C_1 (and α if not equal to 1) so that the level ℓ expectation of bias reduction is approximately $C_1 2^{-\alpha\ell}$
3. Use regression on the three points to estimate C_2 (and β if not equal to 1) so that the level ℓ variance is approximately $C_2 2^{-\beta\ell}$. The variance of the MLMC estimator is therefore $\sum_0^L C_2 2^{-\beta\ell}/N_l$
4. Choose level L large enough to control the bias of the final EVPPI estimator, which is $C_1 2^{-\alpha L} \leq \varepsilon/2$
5. Choose N_ℓ for each level ℓ to minimise the total computational cost ($\sum_0^L N_l 2^\ell$) while achieving RMSE ε, which requires knowledge of the constants C_2 and β. This minimisation can be done by constrained optimisation or by trial and error.

The details of these calculations are given in [20, 21] and illustrated in the supplementary code in section 7.5 of this paper.

Two additional refinements of the MLMC method are explained in the appendix 7.1.2 and

7.1.3. The first is a method of arranging the random samples of X and Y to ensure a higher correlation between successive estimators $\hat{e}_{\ell-1}(N), \hat{e}_\ell(N)$ for which it was shown $\beta = 1.5$ [21, 41]. The second is an alternative method of constructing the series for MLMC estimator, and is preferable when the EVPPI is large compared with the EVPI.

7.1.2 Further details of the antithetic variable approach to the level estimator

In this appendix we discuss how to enable $e_{\ell-1}^{(n)}$ and $e_\ell^{(n)}$ have a higher correlation by utilising all the inner samples. For $e_\ell^{(n)}$ we need to generate 2^ℓ samples of $Y^{(n,m)}$ conditional on $X^{(n)}$. That is

$$e_\ell^{(n)} = \max_{d\in D} \frac{1}{2^\ell}\sum_{m=1}^{2^\ell} f_d(X^{(n)}, Y^{(n,m)}) - \max_{d\in D}\frac{1}{2^\ell N}\sum_{i=1}^{N}\sum_{m=1}^{2^\ell} f_d(X^{(i)}, Y^{(i,m)}).$$

For $e_{\ell-1}^{(n)}$, we use the same $X^{(n)}$ and separate the 2^ℓ samples of $Y^{(n,m)}$ into two sets, both of which have $2^{\ell-1}$ samples, to construct two samples and get the average for the first term and use all the samples for the second term. That is

$$\begin{aligned} e_{\ell-1}^{(n)} &= \frac{1}{2}\left[\max_{d\in D}\frac{1}{2^{\ell-1}}\sum_{m=1}^{2^{\ell-1}} f_d(X^{(n)}, Y^{(n,m)}) + \max_{d\in D}\frac{1}{2^{\ell-1}}\sum_{m=2^{\ell-1}+1}^{2^\ell} f_d(X^{(n)}, Y^{(n,m)})\right] \\ &\quad - \max_{d\in D}\frac{1}{2^\ell N}\sum_{i=1}^{N}\sum_{m=1}^{2^\ell} f_d(X^{(i)}, Y^{(i,m)}) \end{aligned}$$

Then the difference estimator $d_\ell^{(n)}$ becomes

$$\begin{aligned} d_\ell^{(n)} &= \max_{d\in D}\frac{1}{2^\ell}\sum_{m=1}^{2^\ell} f_d(X^{(n)}, Y^{(n,m)}) \\ &\quad - \frac{1}{2}\left[\max_{d\in D}\frac{1}{2^{\ell-1}}\sum_{m=1}^{2^{\ell-1}} f_d(X^{(n)}, Y^{(n,m)}) + \max_{d\in D}\frac{1}{2^{\ell-1}}\sum_{m=2^{\ell-1}+1}^{2^\ell} f_d(X^{(n)}, Y^{(n,m)})\right], \end{aligned} \tag{7.1}$$

Note now that the second term of $e_{\ell-1}^{(n)}$ and $e_\ell^{(n)}$ cancels. If all three terms have the same optimal choice d^*, then $d_\ell^{(n)}$ becomes 0. Theorem 3 in [23] shows that the variance of the new $d_\ell^{(n)}$ is the order of $O(2^{-3\ell/2})$.

7.1.3 MLMC for DIFF

As illustrated in Figures 4(a) and 4(b), it is possible for the EVPPI to be large or small compared with EVPI, respectively. If The EVPI value is known, and it is known that the

EVPPI is close in value to the EVPI, it will require fewer samples to estimate DIFF=EVPI-EVPPI as its smaller magnitude usually leads to a smaller variance. This appendix describes how to construct an estimator for DIFF.

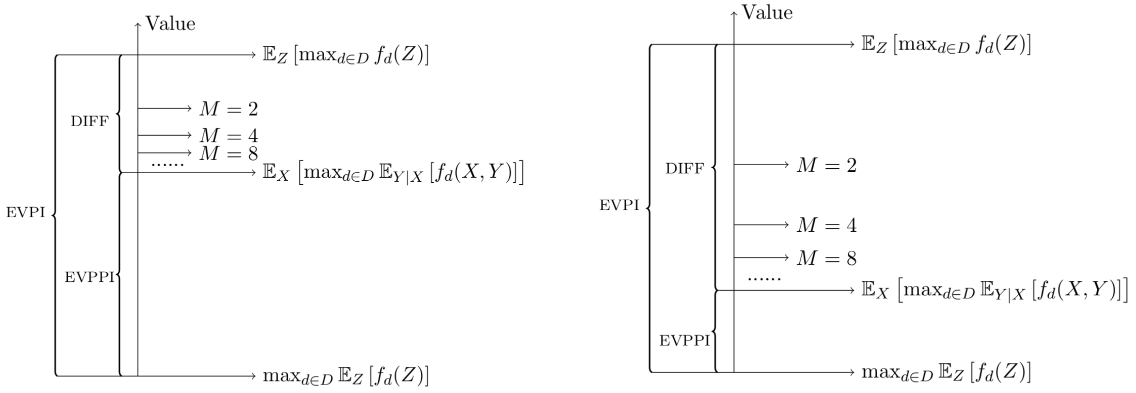

(a) Large EVPPI (b) Small EVPPI

Figure 4: Structure of large or small EVPI

By definition, we have

$$\mathrm{DIFF} = \mathbb{E}_Z\big[\max_{d\in D} f_d(Z)\big] - \mathbb{E}_X\big[\max_{d\in D}\mathbb{E}_{Y|X}[f_d(X,Y)]\big],$$

and the standard nested MC estimator is

$$\widehat{\mathrm{DIFF}}_\ell = \frac{1}{N}\sum_{n=1}^{N}\mathrm{DIFF}_\ell^{(n)}$$

where

$$\mathrm{DIFF}_\ell^{(n)} = \frac{1}{2^\ell}\sum_{m=1}^{2^\ell}\max_{d\in D} f_d(X^{(n)},Y^{(n,m)}) - \max_{d\in D}\frac{1}{2^\ell}\sum_{m=1}^{2^\ell} f_d(X^{(n)},Y^{(n,m)}).$$

For MLMC, we find that $\widehat{\mathrm{DIFF}}_0 = 0$ and the corresponding MLMC estimator of DIFF is

$$\widehat{\mathrm{DIFF}}_\ell^{*}(N_1,N_2,...,N_L) = -\sum_{\ell=1}^{L} d_\ell^{(n)}$$

where $d_\ell^{(n)}$ is the same as (7.1) since the first term of the $\mathrm{DIFF}_\ell^{(n)}$ and $\mathrm{DIFF}_{\ell-1}^{(n)}$ are cancelled out similar to the previous subsection. Compared with (2.7), we get rid of $\hat{e}_0(N_0)$. Therefore, from a practical point of view, it is always easier to estimate a smaller quantity since the variance would be lower. For larger EVPPI as shown in Figure 4(a), it is more reasonable to estimate DIFF instead of estimating EVPPI directly.

	No treatment	CBT	Antidepressant
Total costs (£)	2566.79 (2252.17, 2934.14)	2490.75 (2148.05, 2874.31)	2452.64 (2093.18, 2844.76)
Total QALYs	20.16 (12.53, 27.76)	20.47 (13.16, 27.68)	20.62 (13.49, 27.64)
Incremental costs (£)	0 (0, 0)	-76.04 (-443.73, 287.29)	-114.15 (-537.83, 239.71)
Incremental QALYs	0 (0, 0)	0.30 (-0.12, 1.31)	0.46 (-0.19, 1.80)
Net Benefit (λ=£20,000)	400694 (247991.10, 552672.40)	406863.20 (260751.50, 551144.90)	409983.90 (267368.00, 550330.30)
Incremental NB (λ=£20,000)	0 (0, 0)	6169.19 (-2342.05, 26456.00)	9289.87 (-3672.06, 36367.09)

Table 5: Cost-effectiveness results of the 3-treatment depression toy model.

7.2 Further details of the depression cost-effectiveness toy model

7.2.1 Cost-effectiveness results from the depression toy model

CBT and antidepressants are cheaper than no treatment but antidepressants are the cheapest. CBT and antidepressants confer greater benefits (QALYs) but antidepressants are most effective. Incremental net benefit is highest with antidepressants at a willingness-to-pay threshold (λ) of £20,000. The CEAC indicates that antidepressants have a high probability ($> 75\%$) of being most cost-effective at all realistic willingness-to-pay thresholds.

7.2.2 Details of the 20 treatment option depression model.

In order to explore the complexity that may be encountered in a real cost-effectiveness model, we extended our example from 3 to 20 treatment options. The model structure, outcome costs, and outcome QALYs for each treatment remained the same but treatment effects and costs were randomly generated. However, log odds of relapse and recovery were sampled from a Normal distribution with mean 0 and standard deviation 1 and drug costs were either £500, £1000, £2000, or £3000 with simple rules ensuring treatments with high recovery and low relapse and most expensive, and vice versa (Table 6). The NMA was also extended to 50 trials of up to 5 arms comparing a random selection of the 20 treatments; baseline log odds response and recovery were sampled from Normal with mean 0 and standard deviation 0.25, while numbers recovering or relapsing were sampled from binomials with the appropriate probabilities (Figure 6(a) and Figure 6(b) present network plots for this constructed data).

The cost-effectiveness results are presented in Table 7, with CEACs presented in Figure 7. Treatment 10, which had a high log odds ratio of both recovery (1.85) and relapse (0.81)

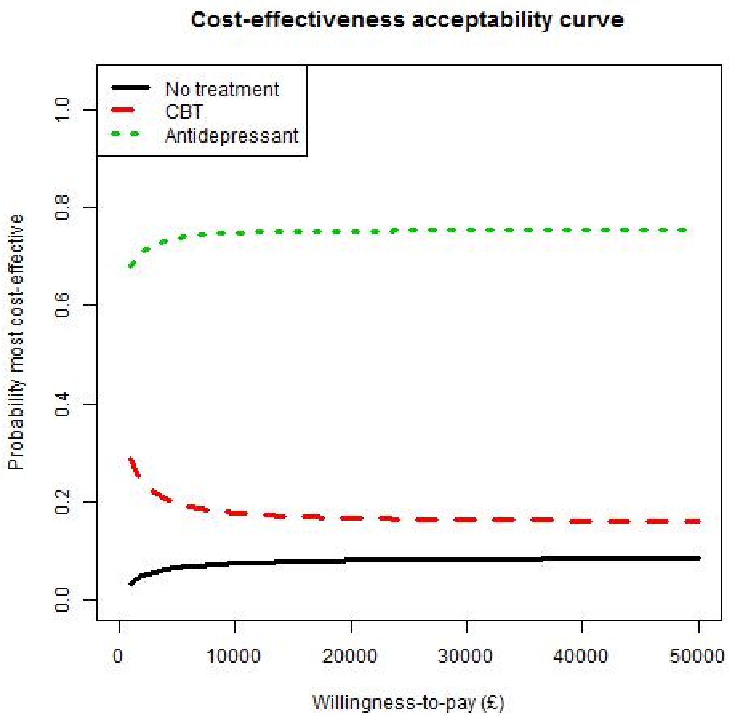

Figure 5: Cost-effectiveness acceptability curve. Each line represents the probability that the corresponding treatment has highest net benefit at each willingness-to-pay threshold.

and a high cost (£2000), had the highest expected incremental net benefit (£14590.49 with 95% Credible Interval (-8706.228, 46266.03)) and greatest probability (> 60%) of being most cost-effective at all willingness-to-pay thresholds.

	Relapse LOR	Recovery LOR	Price (£)
Treatment 1	0.00	0.00	500
Treatment 2	-0.16	0.10	1000
Treatment 3	0.93	1.37	2000
Treatment 4	0.59	0.84	1000
Treatment 5	0.28	0.58	1000
Treatment 6	0.26	-1.97	500
Treatment 7	-0.01	-0.60	1000
Treatment 8	0.72	1.92	2000
Treatment 9	-0.44	-0.26	1000
Treatment 10	0.81	1.85	2000
Treatment 11	-1.43	-0.89	2000
Treatment 12	-0.41	0.56	1000
Treatment 13	-0.93	0.12	1000
Treatment 14	-0.49	0.45	1000
Treatment 15	-1.65	1.15	3000
Treatment 16	-0.36	0.29	1000
Treatment 17	0.40	-0.22	500
Treatment 18	0.58	-0.46	500
Treatment 19	0.13	0.75	1000
Treatment 20	0.49	-2.08	500

Table 6: Treatment effects and costs of the 20-treatment depression model.

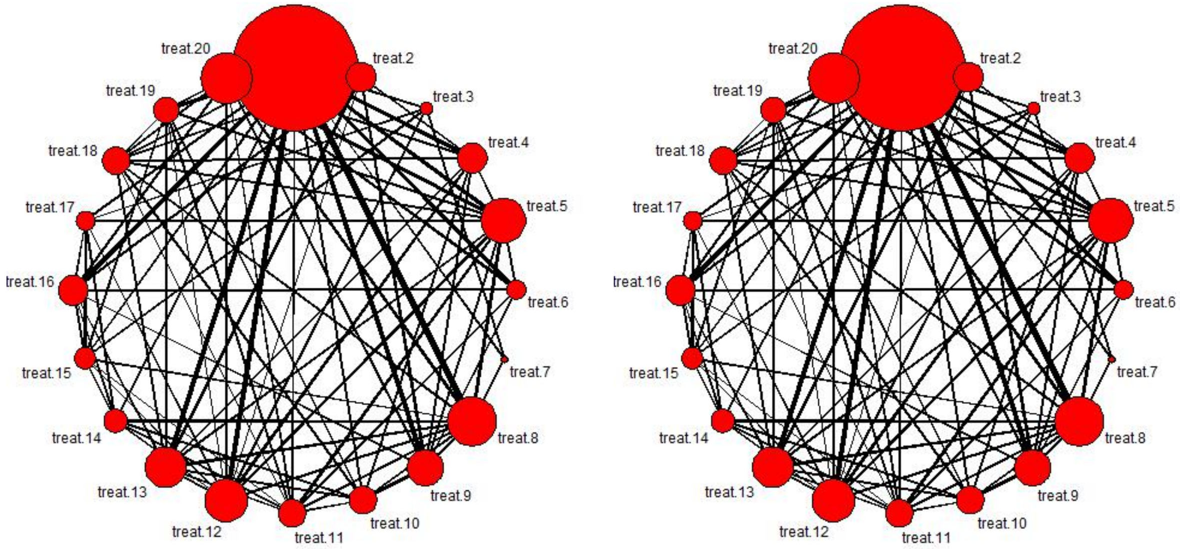

(a) Evidence network for recovery data.

(b) Evidence network for relapse data.

Figure 6: Evidence networks for recovery and relapse data in 20 treatment depression model. Each node represents a treatment being compared. An edge represents a trial comparing the treatments represented by the two connected nodes. If a path can be found between two nodes, the corresponding treatments can be compared by network meta-analysis.

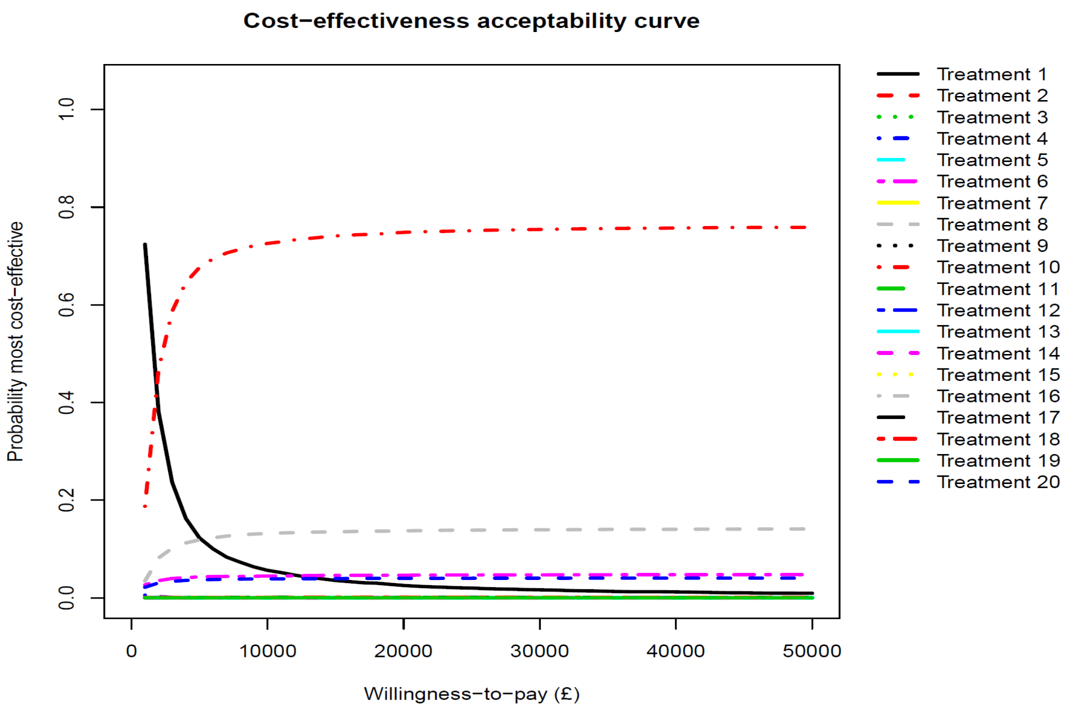

Figure 7: Cost-effectiveness acceptability curves (for any treatment with a probability of being most cost-effective greater than 5% at some threshold) for the 20-treatment depression model.

7.2.3 EVPPI results of the 20 treatment depression model

For comparison, we set the MSE to be 0.25 ($\varepsilon = 0.5$). Table 8 shows the samples needed to achieve a 0.25 MSE. We observe that he computational cost of MC and MLMC in EVPPI for Cost and QALYs are much less than QMC. The reason is that MC and MLMC use DIFF estimator, whereas QMC directly calculates EVPPI. In this case, calculating DIFF is more efficient than EVPPI.

EVPPI	Value	Credible interval	Computational Cost (10^6 samples)		
			MC	MLMC	QMC
$(P^{(d)}_{rec}, P^{(d)}_{rel})$	85.84	[82.45, 89.23]	13.18	27.41	0.32
Cost and QALYs	756.44	[753.05, 759.83]	0.46	0.23	3.2
lor_8 and lor_{10}	83.68	[80.29, 87.07]	28.72	12.37	0.1

Table 8: Comparison of computational cost (measured as number of 10^6 samples required to obtain an MSE of 0.25).

7.3 Quantile-quantile diagnostic plots for BCEA INLA-GP EVPPI estimation

The qq-plots below were produced using the BCEA function **diag.evppi**.

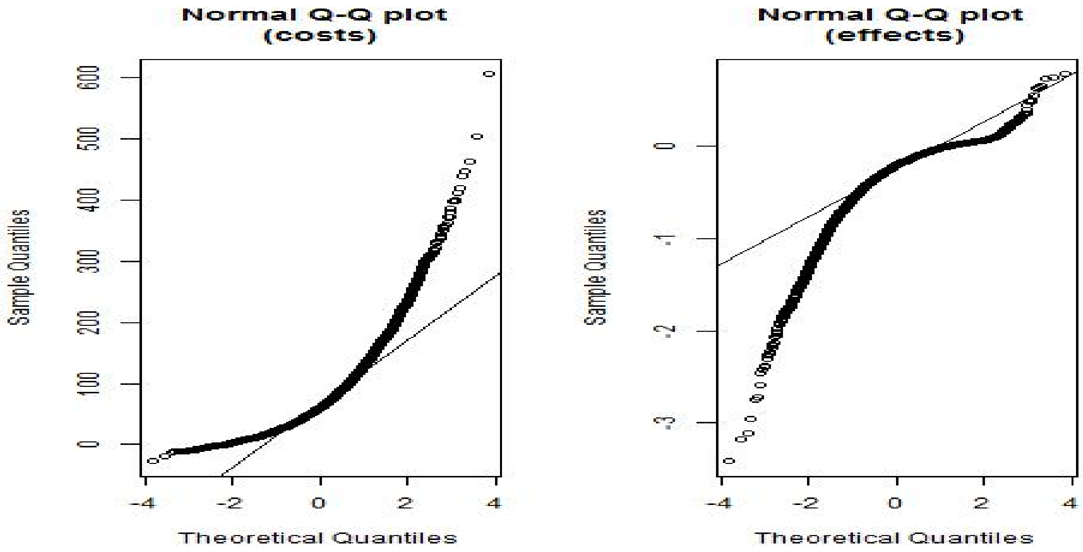

Figure 8: QQ-plots for INLA-GP estimate of EVPPI for costs and QALYs set in depression toy example

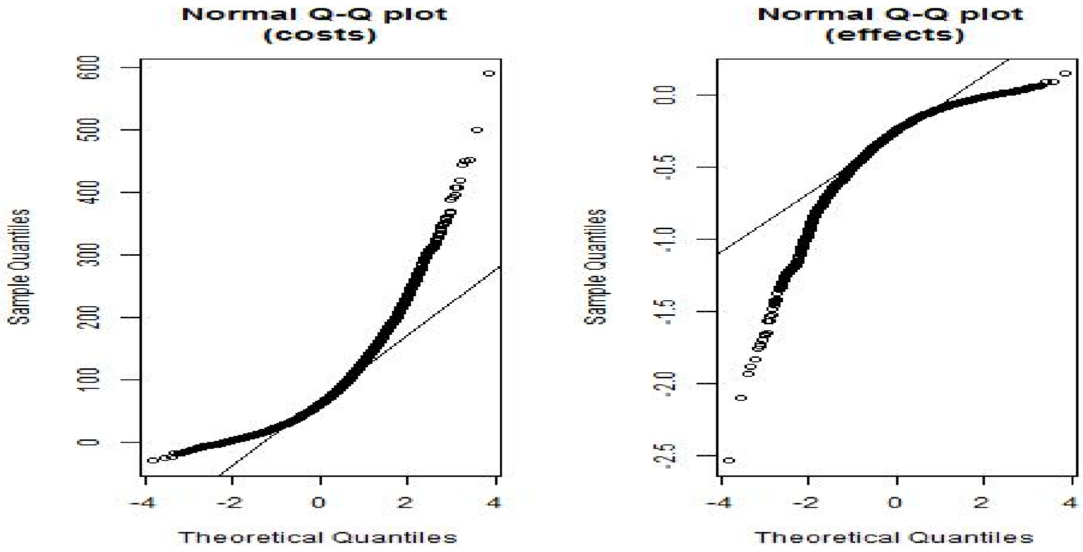

Figure 9: QQ-plots for INLA-GP estimate of EVPPI for probabilities of relapse and recovery in depression toy model

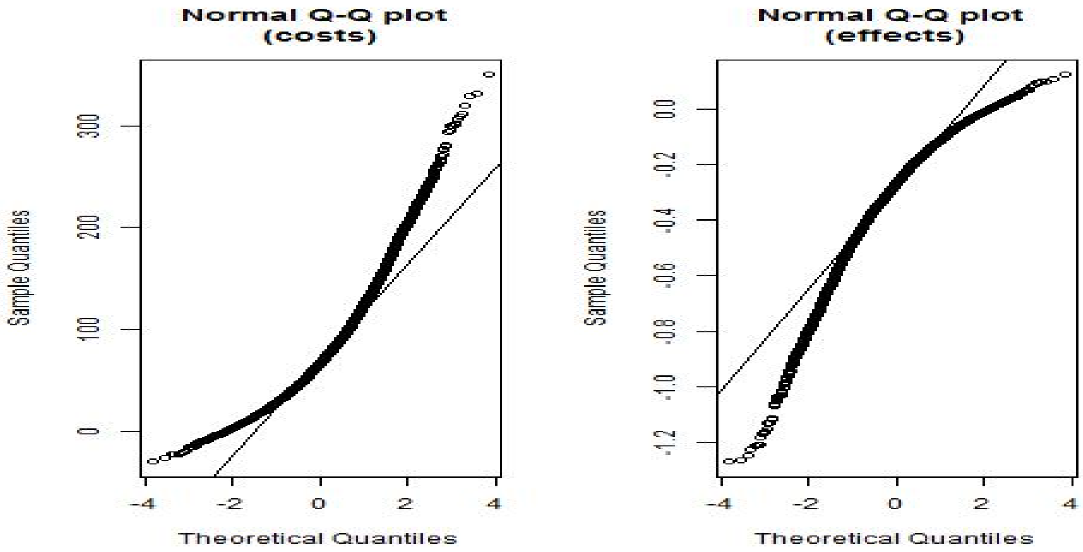

Figure 10: QQ-plots for INLA-GP estimate of EVPPI for CBT relapse and recovery log odds ratios in depression toy model

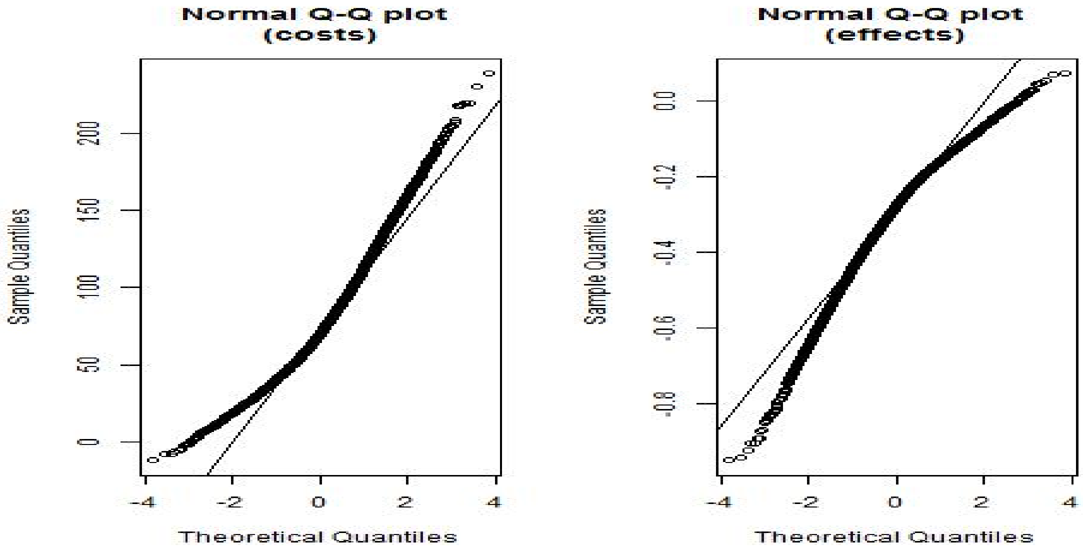

Figure 11: QQ-plots for INLA-GP estimate of EVPPI for antidepressant relapse and recovery log odds ratios in depression toy example

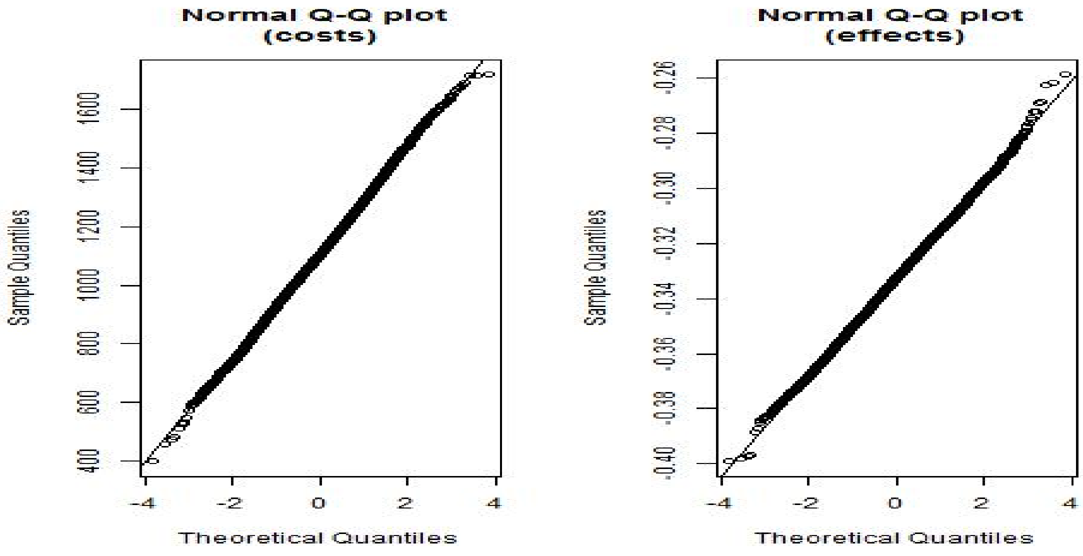

Figure 12: QQ-plots QQ-plots for INLA-GP estimate of EVPPI for simple trial parameter set in DOACs model

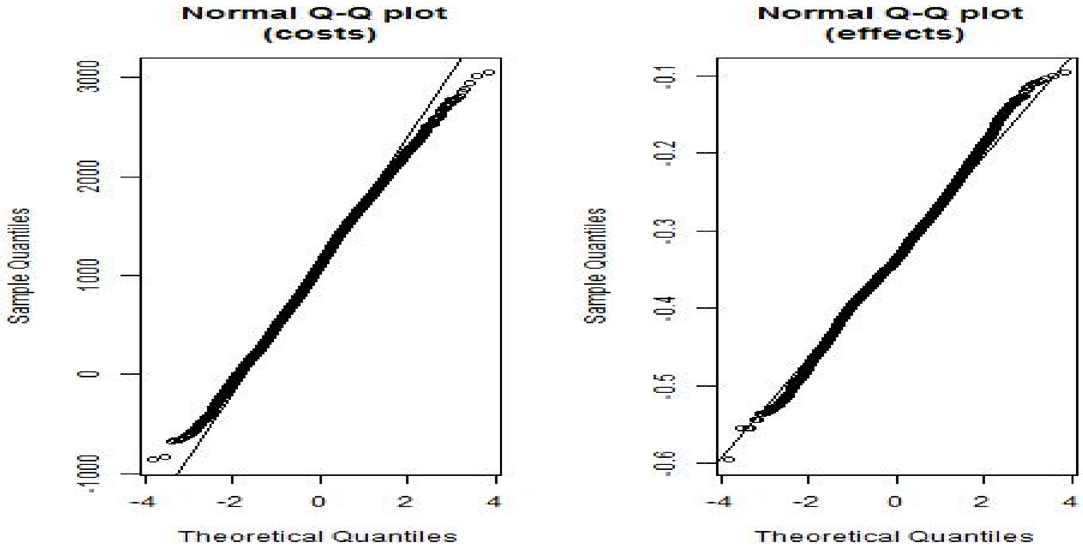

Figure 13: QQ-plots for INLA-GP estimate of EVPPI for log odds ratios in DOACs model

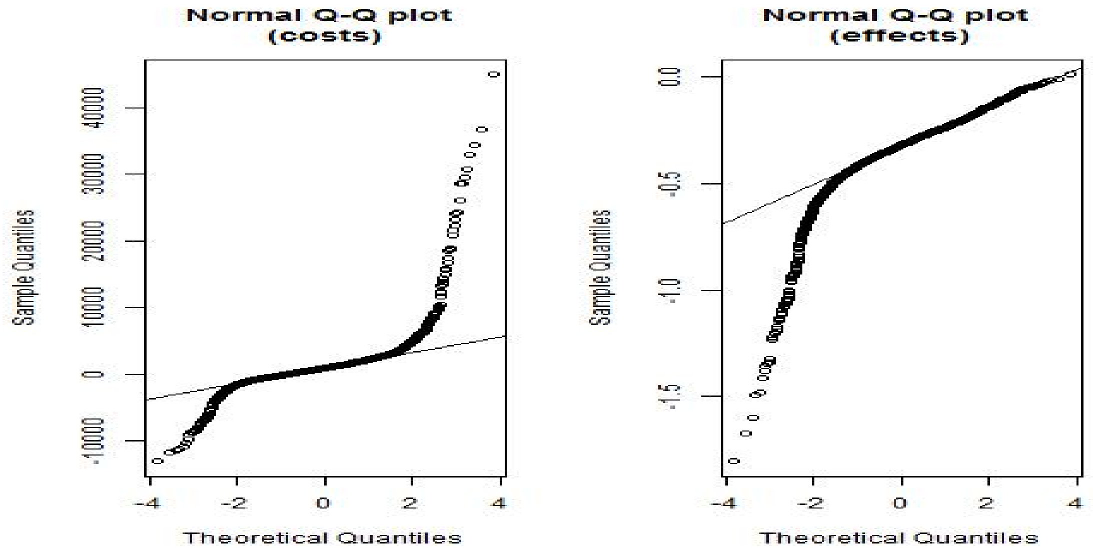

Figure 14: QQ-plots for INLA-GP estimate of EVPPI for all MCMC parameters in DOACs model

7.4 Real, complex health economic model of DOACs in atrial fibrillation

7.4.1 Description of model

In the previous decision tree example, the NB function was simple and quick to evaluate. In this section we present an example where the net benefit function is more complex, so that generating a sample of NBs is more computationally expensive. Thus we might expect that standard nested MC methods require unrealistic sample sizes for a required accuracy. The MCMC sampler required more simulations to characterise the posterior distribution, since a statistical model of higher dimension was used, and the sampled chains were more strongly autocorrelated. This example is a model recently used to compare the cost-effectiveness of DOACs for prevention of stroke in atrial fibrillation, the full details of which have been published [18, 17]. In brief, this was a Markov model with 17 health states, including death, a 3-month cycle length, lifetime horizon, and taking the UK National Health Service perspective. It compared first-line treatment by coumarin with international normalised ratio 2-3 to four DOACs: apixaban 5mg twice daily (BD), dabigatran 150mg BD, rivaroxaban 20mg once daily (OD), and edoxaban 60mg OD in 70 year old adults. Treatment switching from DOAC to coumarin or no treatment or from coumarin to no treatment was modelled. This model included four events with long-term impacts on costs, QALYs, and event risks: ischemic stroke, myocardial infarction (MI), major extracranial bleed, and intracranial haemorrhage (ICH). Health states were defined by which of these states a patient had experienced, giving

the model structure illustrated in Figure 15. In addition, transient ischemic attack (TIA) and systemic embolism (SE) were modelled as transient events with no long-term consequences. Relative effects were informed by a systematic literature review of RCTs comparing DOACs and coumarin. Log hazard ratios relative to coumarin on the seven possible events were estimated using a Bayesian competing risks NMA conducted in OpenBUGS. Log hazard ratios for no treatment compared to coumarin came from a NMA of previously identified RCTs [42]. Log hazards of events on coumarin were estimated using a Bayesian natural history model fit to the coumarin arms of the RCTs [43], thus providing absolute probabilities of events on all possible treatments. MCMC samples from the posterior distributions of these three Bayesian analyses were input directly to the cost-effectiveness model. Costs, utilities, and impact of event history on current risks were estimated using closed form distributions fit to data identified in targeted literature reviews. Full details of all parameters and model assumptions are provided in previous publications [18, 17].

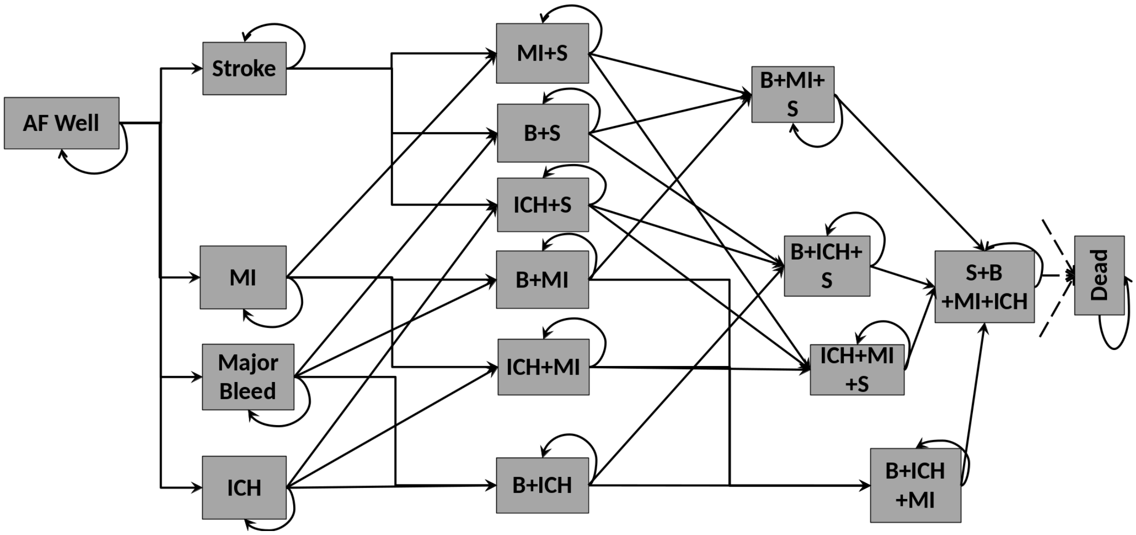

Figure 15: Structure of the DOACs for prevention of stroke in atrial fibrillation cost-effectiveness model. AF=Atrial fibrillation, MI=Myocardial infarction, ICH=Intracranial haemorrhage, B=Major bleed, S=Stroke

In addition to the EVPPI for subsets corresponding to categories of the model parameters (e.g. those generated by MCMC, or treatment switching probabilities), we would like to investigate the potential value of conducing a trial comparing apixaban and dabigatran (the two best DOACs) with a coumarin control arm. We consider two such designs. First is a simple trial that records only the log hazard ratios of apixaban and dabigatran compared to coumarin. Second is a complex trial that records these hazard ratios but also gathers evidence on the absolute probabilities on coumarin, all event and management costs, all event disutilities, all state utilities, and the treatment switching probabilities.

7.4.2 MLMC and QMC results for real, complex health economic model in atrial fibrillation

In summary, the model parameters include 32 where uncertainty is represented by normal random variables, 8 by uniform random variables, 15 by beta distributed random variables, and 42 by 3 sets of MCMC samples (7-dimensional MCMC samples for baseline log hazard ratios, 28-dimensional MCMC samples for log hazard ratios relative to the baseline, and 7-dimensional MCMC samples for log hazard ratios of no treatment).

Table 9 shows the EVPPI estimates computed using the standard MC method, and the relative computational cost required by MLMC and QMC to produce estimates with the same degree of accuracy. MLMC is much more efficient than standard MC when EVPPI is large. In cases where small sample exploration indicated a small or zero EVPPI, we only use standard MC. QMC also works better than standard MC for all the EVPPIs.

Comparing MLMC and QMC, we observe that MLMC performs better for the cases where the parameters are correlated (Simple & Complex Trials), and the QMC works relatively better for the other cases.

7.4.3 Comparison with regression approximation methods on real, complex health economic model in atrial fibrillation

As in section 4.2, we compared our estimates with those provided by GP and INLA-GP, as implemented in SAVI and BCEA, respectively. We conducted comparisons on the large parameter sets where MLMC was necessary. In this case GAM could not be applied as there are too many parameters to identify a regression model without making excessively strong assumptions (such as additivity and linearity) about the relationship between the input parameters and the conditional expected net benefit. Again to compare the relative accuracy of the different methods given the same computational cost, we restricted MC, QMC and MLMC to 50,000 samples and GP and INLA-GP to 7500. Results are presented in Table 10.

This application was more challenging for all methods. The INLA-GP could not estimate for the complex trial. The GP results were severely biased upwards compared to MC, probably due to the poor high-dimensional regression approximation. In addition, they lack face validity as the parameters included in the Loghr MCMC case are a subset of those in the all MCMC case, so the loghr MCMC case should not have a higher EVPPI. The INLA-GP estimates also disagreed with the reference results for all methods, with the possible exception of the MCMC set. However, a poor fit of the underlying regression model was only identified by diagnostic plots in the All MCMC case. The MLMC and QMC methods using only 50,000 samples gave estimates close to the MC reference value, and had RMSEs lower than standard MC.

Again testing testing QMC for estimating total EVPI, we found the EVPI and standard deviation were 416.79 and 5.38 using standard MC and 418.12 and 2.41 using QMC. As in the depression example, this represented reduced bias and variance.

7.5 Reference to GitHub repository with R code

The code discussed in Section 7.5.1 is provided in a supplementary zip file but is also available at https://github.com/Bogdasayen/mlmc_evppi_demo

The depression toy, AF model and MLMC/QMC algorithm code are provided in the GitHub repository located at https://github.com/Bogdasayen/Example_MLMC_and_QMC_for_EVPPI For these models, we briefly point to relevant files below but refer to the repository for complete details.

7.5.1 MLMC demo

We provide the source code mlmc_code_demo_4.rmd along with mlmc.R and mlmc.test.R in a supplemental file. These are also available at https://github.com/Bogdasayen/mlmc_evppi_demo. As noted in the acknowledgements, the mlmc.R and mlmc.test.R code was developed by Mike Giles, Louis Aslett, and Tigran Nagapetyan [39]. This code has been used previously for published MLMC applications [40].

7.5.2 MLMC code for depression toy and AF model

The R implementation of the MLMC algorithm is available in the MLMC package. The main task for a user is to construct the EVPPI level estimator (7.1) or DIFF level estimator (**??**). The depression toy model described in Section 3.1 is provided in the repository Bogdasayen/Example_MLMC_and_QMC_for_EVPPI/ToyModel-MLMC The “ReadMeFirst.pdf” file includes a full description of the code.

In addition the DOAC MLMC code is provided in the repository Bogdasayen/Example_MLMC_and_QMC_for_EVPPI/DOAC-MLMC The model description and summary of the numerical results are in `NOACS_math.pdf` To run the MLMC code, please

1. Run `NOAC.AF.model.main.3.R` to load necessary files and data.
2. Run `MLMC_Core.R` to do a simple test locally.
3. Run `Parellel_Core.R` to run the code using parallel computing.

7.5.3 QMC code for depression toy model

To implement QMC estimate, we start from generating Sobal sequences for uniform distributed random variables U. Then, for a random variable X with cumulative distribution function (CDF) Φ, we get X by $X = \Phi^{-1}(U)$. To calculate EVPPIs, we only need the inverse CDFs for normal distribution qnorm.R and β distrubution, which is implemented in sfunc.R[1].

QMC for the depression toy model is provided in the repository `Bogdasayen/Example_MLMC_and_QMC_for_EVPPI/ToyModel_QMC/` The "ReadMeFirst.pdf" file includes a full description of the code.

In addition, the DOAC QMC code is in the repository `Bogdasayen/Example_MLMC_and_QMC_for_EVPPI/DOAC_QMC/` Note: The only code needed for user to run is `NOAC_QMC_main.R` To run the QMC code, please

1. Open `NOAC_QMC_main.R`, change the baseline.directory
2. Run `NOAC_QMC_main.R`
3. See examples of running EVPPI at the end of `NOAC_QMC_main.R`

7.6 MLMC and QMC when parameters are sampled using Markov Chain Monte Carlo

As discussed in the introduction, cost-effectiveness decision models often rely on Bayesian evidence synthesis to inform parameters so that the joint distribution for some parameters may be represented by samples from an MCMC simulation. Model inputs based on MCMC samples cause three problems for MLMC and QMC estimation. Firstly, if the parameters estimated by MCMC are highly correlated then the number of MCMC samples needed for reliable inference may be large, due to inefficiencies with the MCMC sampler. We address this issue for both MLMC and QMC in Section 7.6.1. Secondly, because MCMC only provides samples and not a closed form representation of the joint distribution, we cannot form the inverse distribution which is needed for QMC. We address this issue Section in 7.6.1. Thirdly, because MCMC does not provide a closed form posterior distribution, we cannot sample from conditional distributions, which are needed for EVPPI estimation whether performed by standard nested MC, MLMC, or QMC. We address this by using multivariate approximations to the posterior distribution is described in Section 7.6.2.

[1]This code is written by Thomas Lumley, for details see https://stat.ethz.ch/pipermail/r-help/2002-January/017624.html.

7.6.1 Random selection of MCMC samples for MLMC and QMC

To reduce the computational expense of generating MCMC samples, we first generate a sufficiently large number N_c of MCMC samples in advance to store in a data set, and randomly select samples from it. Our motivation is that we can then use uniformly distributed random variables for sampling, rather than needing further expensive MCMC. For MLMC, when constructing the level ℓ bias estimator $\hat{d}_\ell(N_\ell)$ we generate 2^ℓ uniform random samples $U^{(n)}$ in $[0,1]$ and choose corresponding MCMC samples with index $\lfloor N_c U^{(n)} \rfloor$ (the largest integer smaller than or equal to $N_c U^{(n)}$). This ensure the same samples in the level ℓ and $\ell - 1$ estimators.

For QMC, the idea is to sort the stored MCMC samples first, and QMC points denote the corresponding position in the sorted sequence. This has the added advantage that it removes the need for an inverse of the posterior distribution. For high dimensional problems, we use an efficient approach to sample a uniform distribution on a large empirical dataset. Given a large enough MCMC sample, we further select the samples we will use in the calculation from the MCMC sample pool. We apply QMC on the random selection. For details on how exactly to apply QMC on random selection, please refer to [44]. This algorithm uses samples from the Uniform distribution to repeatedly bisect the MCMC sample space until only a single sample is found (technical name is recursive spectral bisection algorithm for graph partitioning [45]). The order and elements of X on which to sort is determined by the first two dimensions from principal components analysis (PCA). Using only the first two elements was found to be sufficient for the applications in Section 3, and captured the majority of the impact of the variation of our parameters on the net benefit, and thus the impact on EVPPI. However, if many elements of X had high weights, indicating that the PCA cannot identify two significant dimensions, this approach would be not suitable.

7.6.2 Multivariate approximation of MCMC to obtain conditional distributions

For EVPPI estimation, we need to sample from the distribution of Y conditionally on a specific value of $X^{(n)}$. However this is challenging when X and Y are correlated and their joint distribution is only known through a MCMC sample. In theory, we simply need to set the value of $X^{(n)}$ to be fixed and rerun the MCMC simulation to get $Y^{(n,m)}$. However, this is highly inefficient, since a separate burn-in is required for each individual sample of $X^{(n)}$.

To solve this problem, we compute the mean μ and covariance matrix Σ of the MCMC samples Z, and use the multivariate normal distribution (MVN) $\mathcal{N}(\mu, \Sigma)$ to approximate the posterior distribution. Then the distribution of $Y^{(n,m)}$ conditional on $X^{(n)}$ is known to be another MVN [46]. The benefit is that the random variable we need to generate is Normally distributed and we can apply our MLMC and QMC formulations.

Note that, the MVN approximation is only suitable for parameters whose joint posterior is a symmetric unbounded distribution. Parameters such as hazard ratios or odds ratios will

need to be log transformed to achieve this, as we do in the examples in the following sections. Heavy tailed distributions may be better represented by some other closed form multivariate distribution, such as multivariate t-distribution. However, our approach to MCMC will only work if a transformation to some closed form multivariate distribution (with closed form conditional distributions) is possible.

7.7 MVN vs MVT

The conventional version of the p-dimensional multivariate t (MVT) distribution $X \sim t_p(\mu, \Sigma, \nu)$, with mean μ, scale matrix Σ (generally not the covariance of X), and degrees of freedom ν (which determine the thickness of the tail), has the probability density function

$$f(x) = \frac{\Gamma\{(\nu+p)/2\}}{\Gamma(\nu/2)(\nu\pi)^{p/2}|\Sigma|^{1/2}}\{1 + \nu^{-1}(x-\mu)^T\Sigma^{-1}(x-\mu)\}^{-(\nu+p)/2}. \tag{7.2}$$

The conditional distribution of the multivariate t distribution also follows the multivariate t distribution. Assuming $X = (X_1, X_2)_{p_1,p_2}$, we can define

$$\begin{aligned} \mu_{2|1} &= \mu_2 + \Sigma_{21}\Sigma_{11}^{-1}(X_1 - \mu_1) \\ \Sigma_{22|1} &= \Sigma_{22} - \Sigma_{21}\Sigma_{11}^{-1}\Sigma_{12} \\ d_1 &= (X_1 - \mu_1)^T\Sigma_{11}^{-1}(X_1 - \mu_1) \end{aligned}$$

and then the conditional distribution of X_2 given X_1 is

$$X_2 \mid X_1 \sim t_{p_2}\left(\mu_{2|1},\ \frac{\nu + d_1}{\nu + p_1}\Sigma_{22|1},\ \nu + p_1\right)$$

Compared with the multivariate normal distribution, the MVT can capture the feature of the thick tail and may give better approximation of the covariance structure of the MCMC samples. Here is the comparison results between the two distributions of the EVPPI for DOAC simple trial using standard MC with 1024 outer samples and 128 inner samples:

	EVPPI	EVPPI sd	Bias	Credible interval
Multivariate Normal	184.51	16.01	3.85	[152.23, 216.78]
Multivariate t distribution	189.13	16.89	3.24	[155.42, 222.83]

Table 11: EVPPI value 1024*128

from which we can see that the credible interval of MVT is consistent with the results when using multivariate normal distribution.

	Total costs	Total QALYs	Incremental costs	Incremental QALYs	Net Benefit	Incremental NB
Treatment 1	2956.657 (2716.0, 3197.1)	20.22 (12.7, 27.7)	0 (0, 0)	0 (0, 0)	401453.1 (252603, 551490.9)	0 (0, 0)
Treatment 2	3449.322 (3208.0, 3689.9)	20.24 (12.8, 27.7)	492.66 (482.1, 498.8)	0.029 (-0.012, 0.107)	401541.1 (253699.6, 550615.2)	88.0 (-746.9, 1672.0)
Treatment 3	4342.823 (4094.0, 4592.8)	20.67 (13.7, 27.5)	1386.16 (1288.5, 1455.1)	0.452 (-0.215, 1.38)	409123.7 (271047.1, 547659.8)	7670.6 (-5692.4, 26406.3)
Treatment 4	3394.855 (3150.8, 3638.7)	20.46 (13.2, 27.6)	438.19 (382.86, 476.54)	0.245 (-0.114, 0.753)	405924.7 (262204.9, 549397.4)	4471.6 (-2719.8, 14672.2)
Treatment 5	3422.529 (3181.2, 3662.5)	20.35 (13.0, 27.6)	465.87 (433.91, 487.43)	0.135 (-0.0610, 0.4232)	403691 (257802.8, 550271.7)	2237.9 (-1680.1, 8042.3)
Treatment 6	2994.332 (2750.6, 3237.4)	20.07 (12.4, 27.7)	37.67 (13.8, 74.4)	-0.149 (-0.469, 0.0673)	398434.7 (245141.4, 552521.3)	-3018.3 (-9446.1, 1303.0)
Treatment 7	3477.776 (3236.4, 3719.0)	20.13 (12.5, 27.7)	521.11 (507.6, 541.9)	-0.0834 (-0.262, 0.037)	399263.4 (247884.1, 551555.2)	-2189.6 (-5770.9, 235.8)
Treatment 8	4270.117 (4008.8, 4531.8)	20.96 (14.3, 27.5)	1313.46 (1169.9, 1423.1)	0.740 (-0.349, 2.21)	414947.7 (281912.9, 546828.5)	13494.6 (-8262.5, 43019.6)
Treatment 9	3464.967 (3223.3, 3705.6)	20.18 (12.6, 27.7)	508.31 (502.7, 517.2)	-0.0329 (-0.107, 0.0153)	400285.6 (250357.9, 551439.2)	-1167.4 (-2656.7, -203.8)
Treatment 10	4256.8 (3995.6, 4522.0)	21.01 (14.4, 27.5)	1300.14 (1149.1, 1417.0)	0.794 (-0.371, 2.37)	416043.5 (283951.5, 546673.7)	14590.4 (-8706.2, 46266.0)
Treatment 11	4481.95 (4239.8, 4723.1)	20.12 (12.5, 27.7)	1525.29 (1509.3, 1549.7)	-0.100 (-0.316, 0.0452)	397923.8 (245962.3, 550628.6)	-3529.2 (-7869.5, -618.2)
Treatment 12	3419.747 (3175.8, 3659.0)	20.36 (13.0, 27.6)	463.09 (427.6, 486.3)	0.145 (-0.066, 0.456)	403904.2 (258407.7, 550235.4)	2451.1 (-1791.7, 8693.1)
Treatment 13	3454.433 (3214.4, 3694.1)	20.22 (12.7, 27.7)	497.77 (490.9, 502.5)	0.00855 (-0.0132, 0.0490)	401126.4 (252442.7, 550957.8)	-326.6 (-767.6, 487.4)
Treatment 14	3432.998 (3191.5, 3673.1)	20.31 (12.9, 27.6)	476.34 (453.1, 491.2)	0.0932 (-0.0405, 0.295)	402841 (256229.7, 550297.2)	1387.8 (-1288.1, 5446.3)
Treatment 15	5361.133 (5112.2, 5608.3)	20.59 (13.5, 27.6)	2404.47 (2320.1, 2463.3)	0.3760 (-0.163, 1.14)	406570 (266626.8, 547233.4)	5116.9 (-5666.6, 20639.7)
Treatment 16	3437.581 (3196.8, 3676.8)	20.29 (12.9, 27.6)	480.92 (461.0, 493.3)	0.0750 (-0.0333, 0.241)	402473.2 (255667.2, 550211.3)	1020.1 (-1147.8, 4357.3)
Treatment 17	2963.146 (2722.1, 3203.6)	20.19 (12.7, 27.7)	6.48 (1.8, 14.2)	-0.0254 (-0.0858, 0.0109)	400936.7 (251155.1, 551836.7)	-516.3 (-1726.7, 213.0)
Treatment 18	2972.343 (2731.7, 3212.8)	20.15 (12.6, 27.7)	15.6 (5.7, 31.1)	-0.0617 (-0.195, 0.0270)	400201.6 (249608.4, 552002.6)	-1251.4 (-3941.0, 527.4)
Treatment 19	3411.843 (3168.7, 3651.9)	20.39 (13.1, 27.6)	455.18 (413.1, 483.1)	0.177 (-0.0803, 0.551)	404543.8 (259755.6, 549912.4)	3090.7 (-2054.1, 10598.3)
Treatment 20	2994.37 (2750.6, 3237.6)	20.07 (12.4, 27.7)	37.71 (13.8, 74.2)	-0.149 (-0.469, 0.066)	398432.8 (245143.9, 552534.5)	-3020.2 (-9461.8, 1287.2)

Table 7: Cost-effectiveness results from the 20-treatment depression toy example.

EVPPI	Value	M	RMSE	Bias	Sd	Computational Cost (10^5 samples)		
						MC	MLMC	QMC
Simple Trial	196	2048	1.23	0.25	1.20	2144	68.63	–
Complex Trial	273	256	1.23	0.25	1.20	157	33.78	89.54
All cost	0	256	0.91	0.91	0	2.62	–	2.62
Switch probability	0	128	0.01	0.01	0	1.31	–	1.31
All MCMC	348	256	1.23	0.25	1.20	139.9	24.47	31.72
Baseline MCMC	0	128	0.79	0.57	0.54	1.31	–	0.97
Loghr MCMC	286	256	1.23	0.25	1.20	317.1	31.85	21.21
No treatment MCMC	0	128	1.05	1.05	0	1.31	–	1.31
All Utility	0	256	0.70	0.70	0	2.62	–	2.62

Table 9: EVPPI of the DOACs model: comparison of computational cost (measured in units of 10^5 samples). QMC couldn't give an estimate with ε^2 MSE within a reasonable time due to the large number of inverse normal and beta function evaluations. MLMC couldn't provide estimates for EVPPI in reasonable time when values are nearly 0.

Parameters (size)	MC reference (RMSE) (Bias, SE)	MC (RMSE) (Bias, SE)	QMC (RMSE) (Bias, SE)	MLMC (RMSE) (Bias, SE)	GP (RMSE) (Bias, SE)	INLA-GP
Simple Trial (14)	196 (1.23) (0.25, 1.20)	201.93 (52.70) (11.63, 51.40)	192.02 (17.14) (11.63, 12.59)	194.80 (23.25) (11.63, 20.12)	406.72 (417.07) (412.29, 62.95)	741.09
Complex Trial (51)	273 (1.23) (0.25, 1.20)	248.37 (38.91) (14.52, 36.10)	251.73 (32.40) (14.52, 28.96)	259.40 (29.04) (14.52, 25.12)	742.10 (327.23) (323.21, 51.14)	-
All MCMC (40)	348 (1.23) (0.25, 1.20)	354.22 (13.68) (4.94, 12.76)	361.21 (11.13) (4.94, 9.97)	367.31 (9.87) (4.94, 8.55)	642.95 (140.05) (130.88, 49.83)	256.56
Loghr MCMC (28)	286 (1.23) (0.25, 1.20)	264.49 (16.13) (7.84, 14.10)	266.68 (14.63) (7.84, 12.35)	291.12 (15.68) (7.84, 13.56)	944.69 (522.37) (517.42, 71.76)	551.98

Table 10: Comparison of estimates and uncertainties for EVPPIs in DOACs model. MC reference value is from Table 9, using sufficient samples to achieve pre-specified RMSE. Use 50,000 samples for MC, MLMC and QMC and 7500 samples for GP and INLA-GP. MC reference value is that from Table 9.
